# Chemo-Enzymatic Synthesis of Oligoglycerol Derivatives

**DOI:** 10.3390/molecules21081038

**Published:** 2016-08-09

**Authors:** Abhishek K. Singh, Remi Nguyen, Nicolas Galy, Rainer Haag, Sunil K. Sharma, Christophe Len

**Affiliations:** 1 Department of Chemistry, University of Delhi, Delhi 110007, India; abhikmcdu@gmail.com; 2 Université de Technologie de Compiègne (UTC), CS 60319, Compiègne Cedex 60203, France; remi.nguyen@utc.fr (R.N.); ngaly@laposte.net (N.G.); 3 Institut für Chemie und Biochemie, Freie Universität Berlin, Takustraße 3, Berlin 14195, Germany; rainer.haag@gmx.de

**Keywords:** glycerol oligomers, Novozym 435, chemo-enzymatic, regioselectivity, building blocks

## Abstract

A cleaner and greener method has been developed and used to synthesize 14 different functionalized oligomer derivatives of glycerol in moderate 29%–39% yields over three steps. After successive regioselective enzymatic acylation of the primary hydroxyl groups, etherification or esterification of the secondary hydroxyl groups and chemoselective enzymatic saponification, the target compounds can efficiently be used as versatile building blocks in organic and supramolecular chemistry.

## 1. Introduction

Glycerol is a considered green feedstock due to its bioavailability. An enormous quantity of glycerol is being produced by the oleochemical and biodiesel industry and is used as a base chemical for the production of value added products [1,2,3,4,5,6,7,8,9,10,11,12,13]. It has enormous applications in the food industry, pharmaceutical and personal care preparations. Oligomerization of glycerol and the physical and chemical properties of its oligomers have also been well studied. In particular the low molecular weight oligomers such as di-, tri-, and tetraglycerol are more hydrophilic than higher ones and thus have better solubility in polar solvents. These oligomers are used in personal care formulations for their mild humectant properties and ability to enhance fragrance/flavor impact and longevity. Glycerol oligomers also act as plasticizers in PVA films and starch-based biodegradable thermoplastic compositions. The oligomer derivatives have been explored for various applications, e.g., thickners, emulsifiers, antifogging agents, etc. [14,15,16,17,18,19]. Glycerol oligomers may also be considered as superior building blocks for polymerization or polycondensation reactions in comparison to the monomer as the latter leads to low molecular weight reaction products which have a considerable effect on the properties of the macromolecular compounds. The preparation of polymers based on the conversion of oligomerss into macromolecular compounds is emerging as an interesting line of development in the synthesis of polymers [20,21]. Such a method is associated with the synthesis of oligomers with reactive groups at the ends of the molecules.

In order to provide more extensive data for a wider structure-activity relationship (SAR), analogues having 2-*O*-alkyl and 2-*O*-acyl groups have to be synthesized selectively via full or partial green chemistry. Unfortunately, glycerol and its oligomers have two types of hydroxyl groups having similar pKa and consequently, regioselective differentiation of the primary and secondary hydroxyl groups is difficult. In this regard, a protection-functionalization-deprotection strategy has to be realized. Our preliminary work reported that an enzymatic method using immobilized *Candida antarctica* lipase (Novozym 435) [22,23,24] can distinguish the primary and secondary hydroxyl groups in glycerol and polyglycerol moieties to synthesize a wide variety of polymeric and dendritic architectures for biomedical applications [25,26,27,28,29]. Starting from dimers and trimers of glycerol, regio-selective enzymatic protection of the primary hydroxyl groups by acetylation could be envisaged followed by 2-*O*-alkylation or 2-*O*-acylation of the secondary hydroxyl groups, then followed by deprotection of primary hydroxyls. Herein, we report the synthesis of 14 new building blocks based on diglycerol and triglycerol wherein aromatic/azido groups have been incorporated in the secondary carbon via an ether or an ester so as to provide versatility besides facilitating the monitoring of their reactions and product purification. The presence of aromatic moiety may provide the possibility of additional π-π interactions in the macromolecules and thus controlling the aggregation phenomenon and encapsulation behavior. The incorporation of the azido group and an alkynyl group on the other hand provide a site for the 1,3-dipolar cycloaddition reaction under click conditions [30,31,32].

## 2. Results

**Scheme 1:**
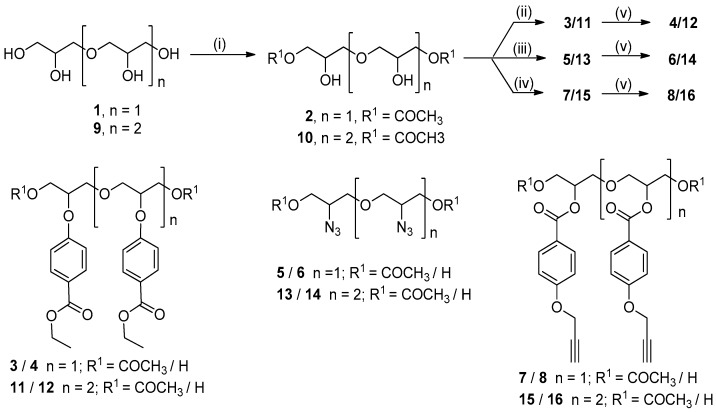
Synthesis of diglycerol/triglycerol based building blocks. *Reagents and Conditions*: (**i**) Novozym 435, vinyl acetate, THF, 6 h, 30 °C; (**ii**) DIAD, TPP, THF, ethyl,4-hydroxybenzoate, 15 h, 40 °C (**iii**) (a) MsCl, DCM, TEA, 2.5 h, 0 to −5 °C. (b) DMF, NaN_3_, 90 °C, 15 h. (**iv**) EDC.HCl, DCM:DMF (4:1), 4-(prop-2-yn-1-yloxy)benzoic acid, 15 h, 30 °C. (**v**) Novozym 435, *n*-butanol, THF, 72 h, 60 °C.

Commercially available diglycerol and triglycerol were subjected to Novozym 435-catalyzed acylation by following the literature procedure [26]. Since the commercially available oligomers are not in pure form and rather are mixtures of glycerol, diglycerol and triglycerol, consequently, a mixture of products is obtained after acylation using Novozym 435 (20 wt %) and vinyl acetate in THF (Scheme 1).

**Figure 1:**
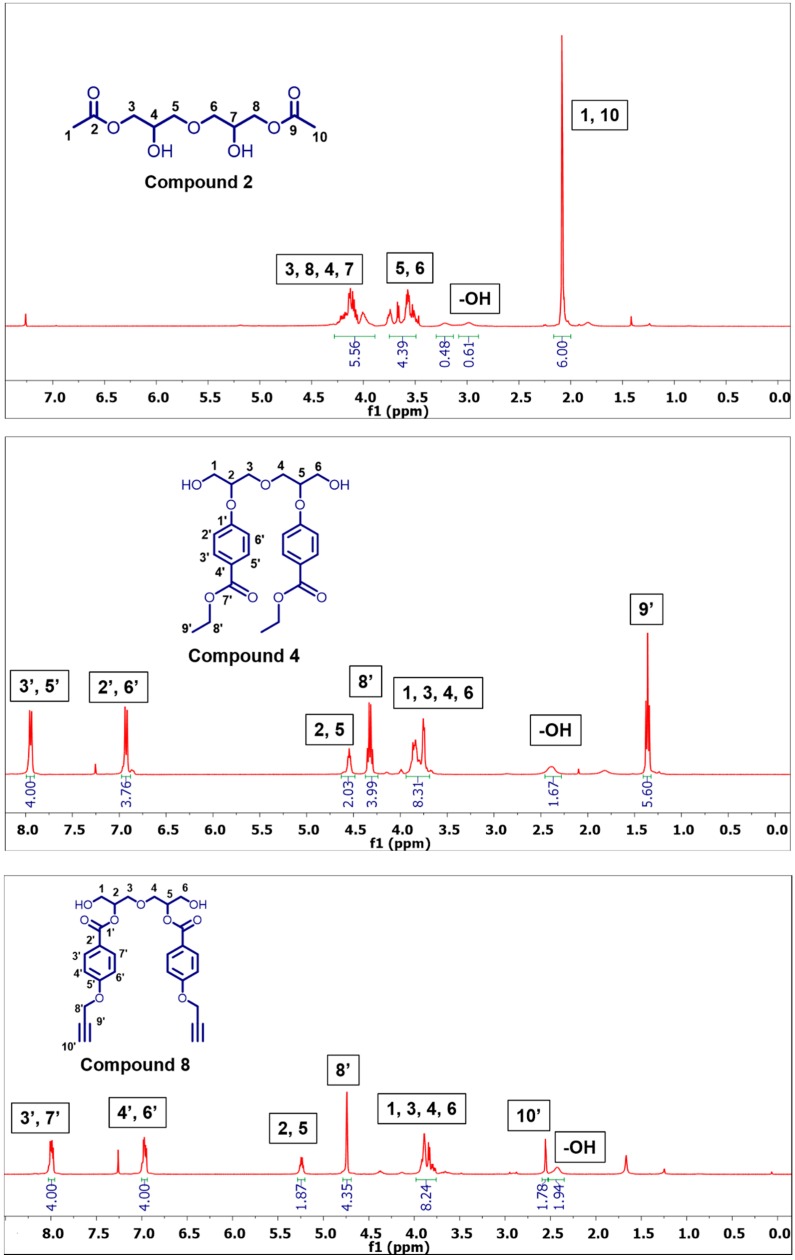
^1^H-NMR spectrum of compound **2**, **4**, and **8**.

**Figure 2:**
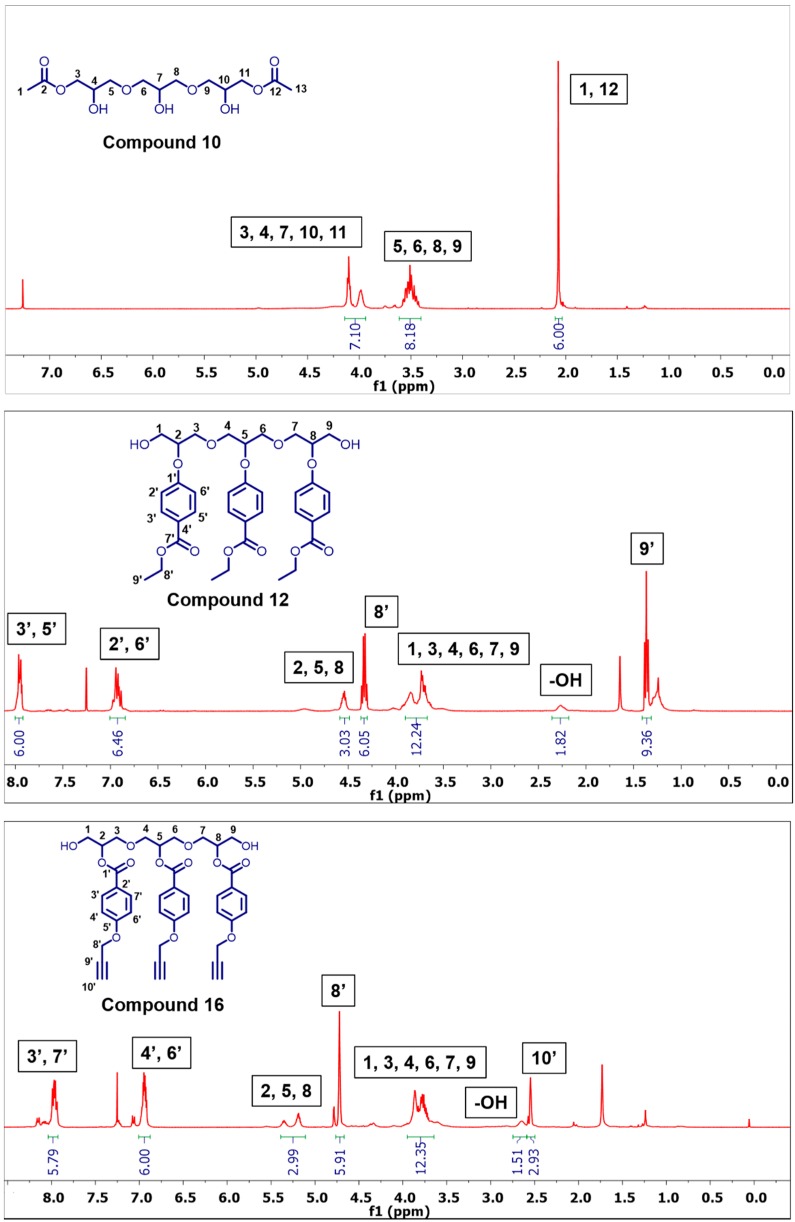
^1^H-NMR spectrum of compound **10**, **12** and **16**.

The reaction progress was monitored by TLC (methanol/chloroform, 1:9, *v*/*v*) and on completion of the reaction the desired diacylated product was purified through column chromatography over silica gel using CHCl_3_/MeOH as eluent It should be noted that compounds **2** and **10** contain two and three stereocenters, respectively. In our hands, both of these compounds were obtained as a mixture of three and seven stereoisomers, respectively and this was confirmed by the observance of the peak multiplicity in the ^13^C-NMR spectrum of compounds **2** and **10** (see Supplementary Materials Figures S1 and S8). This suggests that the enzymatic conditions produced the protected glycerol derivatives regioselectively but not stereoselectively. In the ^1^H-NMR spectrum of compounds **2** and **10**, the methyl protons of the acetyl group appeared at 2.08 ppm, whereas the methylene and methine protons appeared in the range of 3.43 to 4.24 ppm (Figure 1 and Figure 2).

The resulting diacyl oligomers **2** and **10** were further functionalized at the secondary hydroxyl via ether/ester linkage (Scheme 1). The 2-*O*-alkylation of compounds **2** and **10** was realized via Mitsunobu reaction using 4-hydroxybenzoic acid ethyl ester and furnished the two ethers **3** and **11** in 70% and 65% yield, respectively [33]. The 2-*O*-acylation of compounds **2** and **10** using benzoic acid derivative in the presence of EDC gave the corresponding esters **7** and **15** in 75% and 70% yield, respectively [25,34].

Furthermore, the azido group was also incorporated in a stepwise manner i.e., first carrying out mesylation of the secondary hydroxyl group followed by treatment with sodium azide in DMF. The formation of products **5** and **13** was monitored by the appearance of an azide peak in the IR spectra. Our conditions permitted us to produce regioselectively, in the secondary position, the ethers **3** and **11**, esters **7** and **15** and azido derivatives **5** and **13**. Saponification and transesterification were not observed, meaning that the regioselective protection of the primary hydroxyl groups supported the described nucleophilic conditions.

In the next step the resulting diacyl oligomer derivatives **3**, **5**, **7**, **11**, **13**, and **15** were subjected to Novozym 435-catalyzed deacetylation in the presence of excess *n*-butanol in THF [35]. After column chromatography, the target compounds **4**, **6**, **8**, **12**, **14** and **16** were obtained in good yields. A high chemo-selectivity was observed for deacylation of compounds **3**, **7**, **11** and **15**, the aromatic acid ester remained intact and only the selective hydrolysis of aliphatic acid ester was observed. Moreover, in our conditions, no transesterification of the aromatic esters was observed from the secondary hydroxyl group to the primary hydroxyl group. All the compounds were characterized by ^1^H-, ^13^C-NMR and HRMS analysis. The methine proton in all of these compounds undergoes a characteristic chemical shift on functionalization i.e., *O*-alkylation of secondary hydroxyl groups in compounds **4**, **8**, **12** and **16** led to a shift from 3.90–4.02 to 4.50–4.55 ppm (Figure 1 and Figure 2). However, on acylation of the secondary hydroxyl group, the corresponding methine proton undergoes a more significant shift to 5.20–5.44 ppm (Figure 1 and Figure 2). The azido compounds **6** and **14** exhibited a characteristic peak for azide group at about 2100 cm^−1^ in the IR spectrum. While in the compounds **8** and **16** the characteristic acetylinic proton was observed at 2.55–2.56 ppm (Figure 1 and Figure 2) in the ^1^H-NMR spectrum and the acetylinc (-C≡C-) moiety led to the observance of a peak in the rang 2100–2200 cm^−1^ in the IR spectrum.

## 3. Experimental Section

### 3.1. General Information

All the compounds were characterized by their physical and spectral data. Infrared spectra were recorded on a Perkin-Elmer FT-IR Model 9 Spectrophotometer (Perkin-Elmer, Singapore). The ^1^H- and ^13^C-NMR spectra (400 MHz/100.5 MHz) were recorded on Jeol-400 NMR spectrometer (Jeol, Tokyo, Japan) using TMS as an internal standard. The chemical shift values are measured on δ scale and the coupling constant values (*J*) are reported in Hz. The HRMS data were recorded on Agilent-6530, Q-TOF LCMS (Agilent, Singapore).

The diglycerol and triglycerol were obtained from Solvay Bruxelles (Solvay, Bruxelles, Belgium) and Sigma Aldrich (Saint Louis, MO, USA) respectively. All other chemicals and solvents used were purchased from the Spectrochem Pvt. Ltd. (Mumbay, India) and SD Fine Chemicals Pvt. Ltd. (Mumbay, India). All the solvents were distilled prior to their use. Novozym 435 (immobilized *Candida antarctica* lipase) was purchased from Novo Nordisk A/S, Bagsvaerd, Denmark. Reactions were monitored by pre-coated TLC plates (Merck silica gel 60 F254, Darmstadt, Germany), by visualizing the spot in ceric solution stain and iodine. All the compounds were purified by column chromatography using silica gel (100–200 mesh).

### 3.2. Synthesis and Characterization

#### 3.2.1. Synthesis of Oxybis(2-hydroxypropan-3,1-diyl) Diacetate (**2**)

In a 250 mL RB flask diglycerol (**1**, 30.1 mmol) was dissolved in THF (150 mL) followed by the addition of Novozym 435 (20 wt % of monomers). After stirring for 10 min vinyl acetate (69.3 mmol, 5.96 g) was added, the reaction mixture was placed in an incubator shaker at 230 rpm for 6 h at 30 °C. The progress of the reaction was monitored by TLC (methanol–chloroform, 1:9, *v*/*v*). On completion of the reaction, the enzyme was filtered off and washed with methanol. The organic solvent was evaporated under reduced pressure. The obtained crude product was purified by column chromatography using CHCl_3_–MeOH to give the desired compound **2** as a viscous liquid (75%); IR (neat) ν_max_: 3778, 3698, 2983, 1743, 1712 cm^−1^; ^1^H-NMR (CDCl_3_): δ 4.24–3.97 (m, 6H, H-3, H-4, H-7, H-8), 3.67–3.53 (4H, m, H-5, H-6), 3.22 (br s, 1H, OH), 3.00 (br s, 1H, OH), 2.08 (s, 6H, H-1, H-10) ppm; ^13^C-NMR (CDCl_3_): δ 171 (C-2, C-9), 72 (C-5, C-6), 68 (C-4, C-7), 65 (C-3, C-8), 20 (C-1, C-10) ppm; HRMS (positive, acetonitrile): *m*/*z* calcd. for C_10_H_18_O_7_: 250.1053; found [M + Na]^+^: 273.0946.

#### 3.2.2. Synthesis of ((2-Hydroxypropane-1,3-diyl)bis(oxy))bis(2-hydroxypropane-3,1-diyl) Diacetate (**10**)

In a 250 mL RB flask triglycerol (**9**, 30.1 mmol) was dissolved in THF (150 mL) followed by the addition of Novozym 435 (20 wt % of monomers). After stirring for 10 min vinyl acetate (69.3 mmol, 5.96 g) was added, the reaction mixture was placed in an incubator shaker at 230 rpm for 6 h at 30 °C. The progress of the reaction was monitored by TLC (methanol–chloroform, 1:9, *v*/*v*). On completion of the reaction, the enzyme was filtered off and washed with methanol. The organic solvent was evaporated under reduced pressure. The obtained crude product was purified by column chromatography using CHCl_3_-MeOH to give the desired compound **10** as a viscous liquid (70%); IR (neat) ν_max_: 3399, 2880, 1724, 1694 1443 cm^−1^; ^1^H-NMR (CDCl_3_): δ 4.14–4.08 (m, 4H, H-3, H-11), 4.02–3.93 (m, 3H, H-4, H-7, H-10), 3.58–3.43 (m, 8H, H-5, H-6, H-8, H-9), 2.08 (s, 6H, H-1, H-13) ppm; ^13^C-NMR (CDCl_3_): δ 171 (C-2, C-12), 72 (C-5, C-6, C-8, C-9), 69, 68 (C-4, C-7, C-10), 65 (C-3, C-11), 21 (C-1, C-13) ppm; HRMS (positive, acetonitrile): *m*/*z* calcd. for C_13_H_24_O_9_: 324.1420; found [M + Na]^+^: 347.1909.

#### 3.2.3. Synthesis of Diethyl 4,4′-((Oxybis(1-acetoxypropane-3,2-diyl))bis(oxy))dibenzoate (**3**)

To a stirred solution of compound **2** (4.0 mmol), ethyl,4-hydroxybenzoate (8.4 mmol, 1. 39 g) and triphenylphosphine (12.0 mmol, 3.15 g) in THF (20 mL), DIAD (10 mmol, 2.02 g) in THF (5 mL) was added dropwise. The reaction mixture was stirred for 15 h at 40 °C. The progress of the reaction was monitored by TLC (ethyl acetate–petroleum ether, 1:1, *v*/*v*). On completion of the reaction, the reaction mixture was concentrated under reduced pressure and the desired compound was extracted with ethyl acetate (3 × 30 mL). The combined organic layer was dried over anhydrous Na_2_SO_4_ and concentrated in vacuuo. The obtained crude product was purified through column chromatography using petroleum ether-ethyl acetate to give the desired compound **3** as a viscous liquid (70%); IR (neat) ν_max_: 2981, 1740, 1708, 1603, 1506 cm^−1^; ^1^H-NMR (CDCl_3_): δ 7.97–7.94 (m, 4H, H-3′, H-5′), 6.95–6.93 (m, 4H, H-2′, H-6′), 4.69–4.64 (m, 2H, H-4, H-7), 4.34–4.28 (m, 8H, H-3, H-8, H-8′), 3.77–3.70 (m, 4H, H-5, H-6), 2.02 (s, 6H, H-1, H-10), 1.36 (t, 6H, *J =* 8.0, H-9′) ppm; ^13^C-NMR (CDCl_3_): δ 170 (C-2, C-9), 166 (C-7′), 161 (C-1′), 131 (C-3′, C-5′), 123 (C-4′), 115 (C-2′, C-6′), 74 (C-4, C-7), 70 (C-5, C-6), 63 (C-3, C-8), 60 (C-8′), 20 (C-1, C-10), 14 (C-9′) ppm; HRMS (positive, acetonitrile): *m*/*z* calcd. for C_28_H_34_O_11_: 546.2101; found [M + H]^+^: 547.2162.

#### 3.2.4. Synthesis of Diethyl 4,4′-((9-(4-(Ethoxycarbonyl)phenoxy)-2,16-dioxo-3,7,11,15-tetraoxahepta-decane-5,13-diyl)bis(oxy))dibenzoate (**11**)

To a stirred solution of compound **10** (4.0 mmol), ethyl,4-hydroxybenzoate (8.4 mmol, 1.39 g) and triphenylphosphine (12.0 mmol, 3.15 g) in THF (20 mL), DIAD (10 mmol, 2.02 g) in THF (5 mL) was added drop wise. The reaction mixture was stirred for 15 h at 40 °C. The progress of the reaction was monitored by TLC (ethyl acetate–petroleum ether, 1:1, *v*/*v*). On completion of the reaction, the reaction mixture was concentrated under reduced pressure and the desired compound was extracted with ethyl acetate (3 × 30 mL). The combined organic layer was dried over anhydrous Na_2_SO_4_ and concentrated in vacuuo. The obtained crude product was purified through column chromatography using petroleum ether–ethyl acetate to give the desired compound 11 as a viscous liquid (65%); IR (neat) ν_max_: 2992, 1741, 1707, 1603, 1507 cm^−1^; ^1^H-NMR (CDCl_3_): δ 7.97–7.90 (m, 6H, H-3′, H-5′), 6.95-6.85 (m, 6H, H-2′, H-6′), 4.67–4.54 (m, 3H, H-4, H-7, H-10), 4.36–4.31 (m, 6H, H-8′), 4.28–4.25 (m, 4H, H-3, H-11), 3.76–3.66 (m, 8H, H-5, H-6, H-8, H-9), 2.06, 2.02 (s, 6H, H-1, H-13), 1.39–1.35 (m, 9H, H-9′) ppm; ^13^C-NMR (CDCl_3_): δ 170 (C-2, C-12), 166 (C-7′), 161 (C-1′), 131 (C-3′, C-5′), 123 (C-4′), 115 (C-2′, C-6′), 76, 74 (C-4, C-7, C-10), 70 (C-5, C-6, C-8, C-9), 63 (C-3, C-11), 60 (C-8′), 20 (C-1, C-13), 14 (C-9′) ppm; HRMS (positive, acetonitrile): *m*/*z* calcd for C_40_H_48_O_15_: 768.2993; found [M + NH_4_]^+^: 786.3316.

#### 3.2.5. Synthesis of Oxybis(2-azidopropane-3,1-diyl) Diacetate (**5**)

A solution of compound **2** (4.0 mmol) in DCM (30 mL) was cooled under nitrogen atmosphere over ice bath, triethylamine (16.0 mmol, 1.62 g) and methanesulfonyl chloride (12 mmol, 1.37 g) were then added with maintaining the temperature of the reaction mixture at 0 °C. The solution was then stirred at 30 °C for 2.5 h, the progress of the reaction was monitored by TLC (methanol–chloroform, 1:19, *v*/*v*). On completion of the reaction, the salt was filtered and the solvent evaporated under reduced pressure. To the mesylated product (4 mmol) so obtained, sodium azide (24 mmol, 1.56 g) and DMF (30 mL) were added, the reaction mixture was heated at 90 °C for 15 h. The progress of the reaction was monitored by TLC (methanol–chloroform, 1:19, *v*/*v*). On completion of the reaction, DMF was removed under reduced pressure, the residue obtained was extracted with ethyl acetate (3 × 30 mL). The combined organic layer was dried over anhydrous sodium sulphate followed by evaporation of solvent. The crude product was purified by column chromatography using petroleum ether-ethyl acetate to give the desired compound **5** as a viscous liquid (75%); IR (neat) ν_max_: 2922, 2875, 2094, 1739, 1449 cm^−1^; ^1^H-NMR (CDCl_3_): δ 4.24–4.06 (m, 4H, H-3, H-8), 3.78-3.67 (m, 6H, H-4, H-5, H-6, H-7), 2.09, 2.08 (s, 6H, H-1, H-10) ppm; ^13^C-NMR (CDCl_3_): δ 170 (C-2, C-9), 71 (C-5, C-6), 63 (C-3, C-8), 59 (C-4, C-7), 20 (C-1, C-10) ppm; HRMS (positive, acetonitrile): *m*/*z* calcd. for C_10_H_16_N_6_O_5_: 300.1182; found [M + H]^+^: 301.1409.

#### 3.2.6. Synthesis of ((2-Azidopropane-1,3-diyl)bis(oxy))bis(2-azidopropane-3,1-diyl) Diacetate (**13**)

A solution of compound **10** (4.0 mmol) in DCM (30 mL) was cooled under nitrogen atmosphere over ice bath, triethylamine (16.0 mmol, 1.62 g) and methanesulfonyl chloride (12 mmol, 1.37 g) were then added with maintaining the temperature of the reaction mixture at 0 °C. The solution was then stirred at 30 °C for 2.5 h, the progress of the reaction was monitored by TLC (methanol–chloroform, 1:19, *v*/*v*). On completion of the reaction, the salt was filtered and the solvent evaporated under reduced pressure. To the mesylated product (4 mmol) so obtained, sodium azide (24 mmol, 1.56 g) and DMF (30 mL) were added, the reaction mixture was heated at 90 °C for 15 h. The progress of the reaction was monitored by TLC (methanol–chloroform, 1:19, *v*/*v*). On completion of the reaction, DMF was removed under reduced pressure, the residue obtained was extracted with ethyl acetate (3 × 30 mL). The combined organic layer was dried over anhydrous sodium sulphate followed by evaporation of solvent. The crude product was purified by column chromatography using petroleum ether-ethyl acetate to give the desired compound **13** as a viscous liquid (70 %); IR (neat) ν_max_: 2919, 2101, 1737, 1450 cm^−1^; ^1^H-NMR (CDCl_3_): δ 4.24–4.09 (m, 4H, H-3, H-11), 3.82–3.49 (m, 11H, H-4, H-5, H-6, H-7, H-8, H-9, H-10), 2.08 (s, 6H, H-1, H-13) ppm; ^13^C-NMR (CDCl_3_): δ 170 (C-1, C-13), 71 (C-5, C-6, C-8, C-9,), 70 (C-3, C-11), 63 (C-7), 59 (C-4, C-10), 20 (C-1, C-13) ppm; HRMS (positive, acetonitrile): *m*/*z* calcd. for C_10_H_18_O_7_: 399.1615; found [M + NH_4_]^+^: 417.1971.

#### 3.2.7. Synthesis of Oxybis(1-acetoxypropane-3,2-diyl) bis(4-(prop-2-yn-1-yloxy)benzoate) (**7**)

Compound **2** (4.0 mmol) and 4-(prop-2-yn-1-yloxy)benzoic acid (8.4 mmol) were dissolved in a mixture of DCM and DMF in 4:1 ratio (30 mL). The reaction mixture was stirred at 0 °C, then EDC.HCl (10 mmol, 1.91 g) and DMAP (6 mmol, 0.73 g) were added, the reaction mixture was stirred for 15 h at 40 °C. The progress of the reaction was monitored by TLC (petroleum ether–ethyl acetate, 1:1, *v*/*v*). On completion of the reaction, the solvent was removed under reduced pressure and the residue obtained was extracted with ethyl acetate (3 × 30 mL), the organic layer was dried over anhydrous Na_2_SO_4_ and solvent evaporated under reduced pressure. The resulting crude product was purified by column chromatography using petroleum ether–zethyl acetate to give the desired compound **7** as a viscous liquid (75%); IR (neat) ν_max_: 3284, 2932, 2878, 2123, 1712, 1603, 1508 cm^−1^; ^1^H-NMR (CDCl_3_): δ 7.98–7.96 (m, 4H, H-3′, H-7′), 6.98–6.95 (m, 4H, H-4′, H-6′), 5.44–5.38 (m, 2H, H-4, H-7), 4.75–4.74 (m, 4H, H-8′), 4.41–4.29 (m, 4H, H-3, H-8), 3.82–3.70 (m, 4H, H-5, H-6), 2.56 (m, H-10′), 2.03 (s, 6H, H-1, H-10) ppm; ^13^C-NMR (CDCl_3_): δ 171 (C-2, C-9), 165 (C-1′), 161 (C-5′), 132 (C-3′, C-7′), 122 (C-2′), 114 (C-4′, C-6′), 77 (C-9′), 76 (C-10′), 70 (C-4, C-7), 69 (C-5, C-6), 62 (C-3, C-8), 55 (C-8′), 21 (C-1, C-10) ppm; HRMS (positive, acetonitrile): *m*/*z* calcd. for C_30_H_30_O_11_: 566.1788; found [M + NH_4_]^+^: 584.2123.

#### 3.2.8. Synthesis of 2,16-Dioxo-9-((4-(prop-2-yn-1-yloxy)benzoyl)oxy)-3,7,11,15-tetraoxaheptadecane-5,13-diyl bis(4-(prop-2-yn-1-yloxy)benzoate) (**15**)

Compound **10** (4.0 mmol) and 4-(prop-2-yn-1-yloxy)benzoic acid (8.4 mmol) were dissolved in a mixture of DCM and DMF in 4:1 ratio (30 mL). The reaction mixture was stirred at 0 °C, then EDC.HCl (10 mmol, 1.91 g) and DMAP (6 mmol, 0.73 g) were added, the reaction mixture was stirred for 15 h at 40 °C. The progress of the reaction was monitored by TLC (petroleum ether–ethyl acetate, 1:1, *v*/*v*). On completion of the reaction, the solvent was removed under reduced pressure and the residue obtained was extracted with ethyl acetate (3 × 30 mL), the organic layer was dried over anhydrous Na_2_SO_4_ and solvent evaporated under reduced pressure. The resulting crude product was purified by column chromatography using petroleum ether–ethyl acetate to give the desired compound **15** as a viscous liquid (70%); IR (neat) ν_max_: 3286, 2923, 2122, 1714, 1604, 1507 cm^−1^; ^1^H-NMR (CDCl_3_): δ 8.18–7.95 (m, 6H, H-3′, H-7′), 7.26–6.94 (m, 6H, H-4′, H-6′), 5.39–5.24 (m, 3H, H-4, H-7, H-10), 4.80–4.73 (m, 6H, H-8′), 4.37–4.27 (m, 4H, H-3, H-11), 3.90–3.62 (m, 8H, H-5, H-6, H-8, H-9), 2.58–2.50 (m, 3H, H-10′), 2.03 (s, 6H, H-1, H-13) ppm; ^13^C-NMR (CDCl_3_): δ 170 (C-2, C-12), 165 (C-1′), 161 (C-5′), 131 (C-3′, C-7′), 123 (C-2′), 114 (C-4′, C-6′), 77 (C-9′), 76 (C-10′), 70 (C-4, C-7, C-10), 70 (C-5, C-6, C-8, C-9), 62 (C-3, C-11), 55 (C-8′) 20 (C-1, C-13) ppm; HRMS (positive, acetonitrile): *m*/*z* calcd. for C_43_H_42_O_15_: 798.2524; found [M + NH_4_]^+^: 816.2855.

#### 3.2.9. Synthesis of 4,4′-((Oxybis(1-hydroxypropane-3,2-diyl))bis(oxy))dibenzoate (**4**)

Compound **3** (0.91 mmol) was dissolved in THF (1 mL), then *n*-butanol (1 mL) and Novozym 435 (50 wt % of monomer) were added. The reaction mixture was kept in an incubator shaker at 230 rpm for 72 h at 60 °C. The progress of the reaction was monitored by TLC (methanol–chloroform, 1:9, *v*/*v*). On completion of the reaction, the enzyme was filtered off and washed with methanol. The filtrate was concentrated under reduced pressure. The crude product so obtained was purified by column chromatography using CHCl_3_–MeOH to give the desired compound **4** as a viscous liquid (75%); IR (neat) ν_max_: 3440, 2935, 17033, 1603, 1507 cm^−1^; ^1^H-NMR (CDCl_3_): δ 7.95 (d, 4H, *J* = 8.0, H-3′, H-5′), 6.93 (d, 4H, *J* = 8.0, H-2′, H-6′), 4.56–4.54 (m, 2H, H-2, H-5), 4.36–4.30 (m, 4H, H-8′), 4.00–4.72 (m, 8H, H-1, H-3, H-4, H-6), 2.39 (br s, 2H, OH), 1.37 (t, 6H, H-9′) ppm; ^13^C-NMR (CDCl_3_): δ 166 (C-7′), 161 (C-1′), 131 (C-3′, C-5′), 123 (C-4′), 115 (C-2′, C-6′), 77 (C-2, C-5), 70 (C-3, C-4), 62 (C-1, C-6), 61 (C-8′), 14 (C-9′) ppm; HRMS (positive, acetonitrile): *m*/*z* calcd. for C_24_H_30_O_9_: 462.1890; found [M + H]^+^: 463.1964.

#### 3.2.10. Synthesis of Diethyl 4,4′-((((2-(4-(ethoxycarbonyl)phenoxy)propane-1,3-diyl)bis(oxy))bis(1-hydroxypropane-3,2-diyl))bis(oxy))dibenzoate (**12**)

Compound **11** (0.91 mmol) was dissolved in THF (1 mL), then *n*-butanol (1 mL) and Novozym 435 (50 wt % of monomer) were added. The reaction mixture was kept in an incubator shaker at 230 rpm for 72 h at 60 °C. The progress of the reaction was monitored by TLC (methanol–chloroform, 1:9, *v*/*v*). On completion of the reaction, the enzyme was filtered off and washed with methanol. The filtrate was concentrated under reduced pressure. The crude product so obtained was purified by column chromatography using CHCl_3_–MeOH to give the desired compound **12** as a viscous liquid (65%); IR (neat) ν_max_: 3465, 2980, 2928, 1702, 1603, 1507 cm^−1^; ^1^H-NMR (CDCl_3_, 25 °C): δ 7.97–7.94 (m, 6H, H-3′, H-5′), 6.98–6.89 (m, 6H, H-2′, H-6′), 4.58–4.53 (m, 3H, H-2, H-5, H-8), 4.37–4.31 (m, 6H, H-8′), 3.92–3.70 (m, 12H, H-1, H-3, H-4, H-6, H-7, H-9), 2.27 (br s, 2H, OH), 1.39 (m, 9H, H-9′) ppm; ^13^C-NMR (CDCl_3_): δ 166 (C-7′), 161 (C-1′), 131 (C-3′, C-5′), 124 (C-4′), 115 (C-2′, C-6′), 75, 70 (C-2, C-5, C-8), 62 (C-3, C-4, C-6, C-7), 62 (C-1, C-9), 60 (C-8′), 14 (C-9′) ppm; HRMS (positive, acetonitrile): *m*/*z* calcd. for C_36_H_44_O_13_: 684.2773; found [M + NH_4_]^+^: 702.3113.

#### 3.2.11. Synthesis of 3,3′-Oxybis(2-azidopropan-1-ol) (**6**)

Compound **5** (1.6 mmol) was dissolved in THF (1 mL), then *n*-butanol (1 mL) and Novozym 435 (50 wt % of monomer) were added. The reaction mixture was kept in an incubator shaker at 230 rpm for 72 h at 60 °C. The progress of the reaction was monitored by TLC (methanol–chloroform, 1:9, *v*/*v*). On completion of the reaction, the enzyme was filtered off and washed with methanol. The filtrate was concentrated under reduced pressure. The crude product obtained was purified by column chromatography using CHCl_3_–MeOH as an eluent to give the desired compound **6** as a viscous liquid (70%); IR (neat) ν_max_: 3357, 2926, 2978, 2088, 1638, 1464 cm^−1^; ^1^H-NMR (CDCl_3_): δ 3.78–3.65 (m, 10H, H-1, H-2, H-3, H-4, H-5, H-6), 2.45 (br s, 2H, OH) ppm; ^13^C-NMR (CDCl_3_): δ 71 (C-3, C-4), 62 (C-2, C-5), 62 (C-1, C-6) ppm. HRMS (positive, acetonitrile): *m*/*z* calcd. for C_6_H_12_N_6_O_3_: 216.0971; found [M + H]^+^: 217.1044.

#### 3.2.12. Synthesis of 3,3′-((2-Azidopropane-1,3-diyl)bis(oxy))bis(2-azidopropan-1-ol) (**14**)

Compound **13** (1.6 mmol) was dissolved in THF (1 mL), then *n*-butanol (1 mL) and Novozym 435 (50 wt % of monomer) were added. The reaction mixture was kept in an incubator shaker at 230 rpm for 72 h at 60 °C. The progress of the reaction was monitored by TLC (methanol–chloroform, 1:9, *v*/*v*). On completion of the reaction, the enzyme was filtered off and washed with methanol. The filtrate was concentrated under reduced pressure. The crude product obtained was purified by column chromatography using CHCl_3_–MeOH as an eluent to give the desired compound **14** as a viscous liquid (60%); IR (neat) ν_max_: 3390, 2915, 1988, 2105, 1748, 1480 cm^−1^; ^1^H-NMR (CDCl_3_): δ 3.78–3.55 (m, 15H, H-1–H-9) ppm; ^13^C-NMR (CDCl_3_, 25 °C): δ 71 (C-4, C-6), 70 (C-3, C-7), 62 (C-1, C-9), 62, 60 (C-2, C-5, C-8) ppm; HRMS (positive, acetonitrile): *m*/*z* calcd. for C_9_H_17_N_9_O_4_: 315.14.04; found [M + H]^+^: 338.1298.

#### 3.2.13. Synthesis of Oxybis(1-hydroxypropane-3,2-diyl) bis(4-(prop-2-yn-1-yloxy)benzoate) (**8**)

Compound **7** (0.88 mmol) was dissolved in THF (1 mL), then *n*-butanol (1 mL) and Novozym 435 (50 wt % of monomer) were added. The reaction mixture was kept in an incubator shaker at 230 rpm for 72 h at 60 °C. The progress of the reaction was monitored by TLC (methanol–chloroform, 1:9, *v*/*v*). On completion of the reaction, the enzyme was filtered and washed with methanol. The filtrate was concentrated under reduced pressure. The obtained crude product was purified by column chromatography using CHCl_3_–MeOH to give the desired compound **8** as a viscous liquid (70%); IR (neat) ν_max_: 3459, 3289, 2932, 2880, 2123, 1701, 1603, 1508 cm^−1^; ^1^H-NMR (CDCl_3_): δ 8.01–7.98 (m, 4H, H-3′, H-7′), 6.98–6.95 (m, 4H, H-4′, H-6′), 5.26–5.22 (m, 2H, H-2, H-5), 4.74 (m, 4H, H-8′), 3.92–3.73 (m, 8H, H-1, H-3, H-4, H-6), 2.56 (m, 2H, H-10′), 2.43 (br s, 2H, OH) ppm; ^13^C-NMR (CDCl_3_): δ 166 (C-1′), 161 (C-5′), 132 (C-3′, C-7′), 123 (C-2′), 114 (C-4′, C-6′), 77 (C-9′), 76 (C-10′), 73 (C-2, C-5), 70 (C-3, C-4), 62 (C-1, C-6), 55 (C-8′) ppm; HRMS (positive, acetonitrile): *m*/*z* calcd. for C_26_H_26_O_9_: 482.1577; found [M + Na]^+^: 505.1459.

#### 3.2.14. Synthesis of ((2-((4-(Prop-2-yn-1-yloxy)benzoyl)oxy)propane-1,3-diyl)bis(oxy))bis(1-hydroxypropane-3,2-diyl) bis(4-(Prop-2-yn-1-yloxy)benzoate) (**16**)

Compound **15** (0.88 mmol) was dissolved in THF (1 mL), then *n*-butanol (1 mL) and Novozym 435 (50 wt % of monomer) were added. The reaction mixture was kept in an incubator shaker at 230 rpm for 72 h at 60 °C. The progress of the reaction was monitored by TLC (methanol–chloroform, 1:9, *v*/*v*). On completion of the reaction, the enzyme was filtered and washed with methanol. The filtrate was concentrated under reduced pressure. The obtained crude product was purified by column chromatography using CHCl_3_–MeOH to give the desired compound **16** as a viscous liquid (65%); IR (neat) ν_max_: 3286, 2955, 2878, 2123, 1713, 1603, 1508 cm^−1^; ^1^H-NMR (CDCl_3_): δ 8.17–7.94 (m, 6H, H-3′, H-7′), 7.08–6.94 (m, 6H, H-4′, H-6′), 5.39–5.18 (m, 3H, H-2, H-5, H-8), 4.79–4.73 (m, 6H, H-8′), 3.96–3.67 (m, 12H, H-1, H-3, H-4, H-6, H-7, H-9), 2.66 (br s, 2H, OH), 2.59–2.55 (m, 3H, H-10′) ppm; ^13^C-NMR (CDCl_3_): δ 165 (C-1′), 161 (C-5′), 131 (C-3′, C-7′), 123 (C-2′), 114 (C-4′, C-6′), 77 (C-9′), 76 (C-10′), 73 (C-2, C-5, C-8), 70 (C-3, C-4, C-6, C-7), 62 (C-1, C-9), 55 (C-8′) ppm; HRMS (positive, acetonitrile): *m*/*z* calcd. for C_39_H_38_O_13_: 714.2303; found [M + Na]^+^: 737.2198.

## 4. Conclusions

A series of 13 novel glycerol oligomer derivatives **3**–**8** and **10**–**16** have been synthesized using chemo-enzymatic approach that can be used further for the synthesis of supramolecular architectures. The enzyme catalyzed approach exhibits high chemo- and regioselectivity as the primary hydroxyl group can be selectively acylated in the presence of secondary hydroxyl group. The acetyl groups of the hydroxymethyl of polymers **2** and **10** were stable during the acylation, etherification and azidation. Moreover selective enzymatic deprotection of the primary hydroxyl groups was efficient without saponification and trans-esterification of the ester in the secondary hydroxyl group.

## Supplementary Materials

Supplementary materials can be accessed at: http://www.mdpi.com/1420-3049/21/8/1038/s1.

## Abbreviations

The following abbreviations are used in this manuscript:

DMF: *N*,*N*-dimethylformamide
EDC: 1-ethyl-3-(3-dimethylaminopropyl)carbodiimide
FT-IR: Fourier transform infrared
HRMS: high-resolution mass spectrometry
IR: infrared
MeOH: methanol
NMR: nuclear magnetic resonance
pKa: acid dissociation constant
ppm: parts per million
PVA: polyvinyl acetate
Q-TOF LCMS: quadrupoletime of flight liquid chromatography mass spectrometry
RB: round-bottom
rpm: round per min
SAR: structure-activity relationship
THF: tetrahydrofuran
TLC: thin layer chromatography
TMS: tetramethylsilane
