# Supplementary material for: Chemo-Enzymatic Synthesis of Oligoglycerol Derivatives
Source: Molecules. 2016 Aug 9;21(8):1038. doi: 10.3390/molecules21081038 (PMC6273276; doi:10.3390/molecules21081038)
Supplement: Supplementary file 1 [file molecules-21-01038-s001.pdf]

Supplementary Materials: Chemo-Enzymatic Synthesis of Oligoglycerol Derivatives

Abhishek K. Singh, Remi Nguyen, Nicolas Galy, Rainer Haag, Sunil K. Sharma and Christophe Len

Table of Contents

Figure S1: ^{1}H-, ^{13}C-NMR, HETCORE and HRMS spectra of compound **2**
Figure S2: ^{1}H-, ^{13}C-NMR and HRMS spectra of compound **3**
Figure S3: ^{1}H-, ^{13}C-NMR and HRMS spectra of compound **4**
Figure S4: ^{1}H-, ^{13}C-NMR and HRMS spectra of compound **5**
Figure S5: ^{1}H-, ^{13}C-NMR and HRMS spectra of compound **6**
Figure S6: ^{1}H-, ^{13}C-NMR and HRMS spectra of compound **7**
Figure S7: ^{1}H-, ^{13}C NMR and HRMS spectra of compound **8**
Figure S8: ^{1}H-, ^{13}C-NMR and HRMS spectra of compound **10**
Figure S9: ^{1}H-, ^{13}C-NMR and HRMS spectra of compound **11**
Figure S10: ^{1}H-, ^{13}C-NMR and HRMS spectra of compound **12**
Figure S11: ^{1}H-, ^{13}C-NMR and HRMS spectra of compound **13**
Figure S12: ^{1}H-, ^{13}C-NMR and HRMS spectra of compound **14**
Figure S13: ^{1}H-, ^{13}C-NMR and HRMS spectra of compound **15**
Figure S14: ^{1}H-, ^{13}C-NMR and HRMS spectra of compound **16**

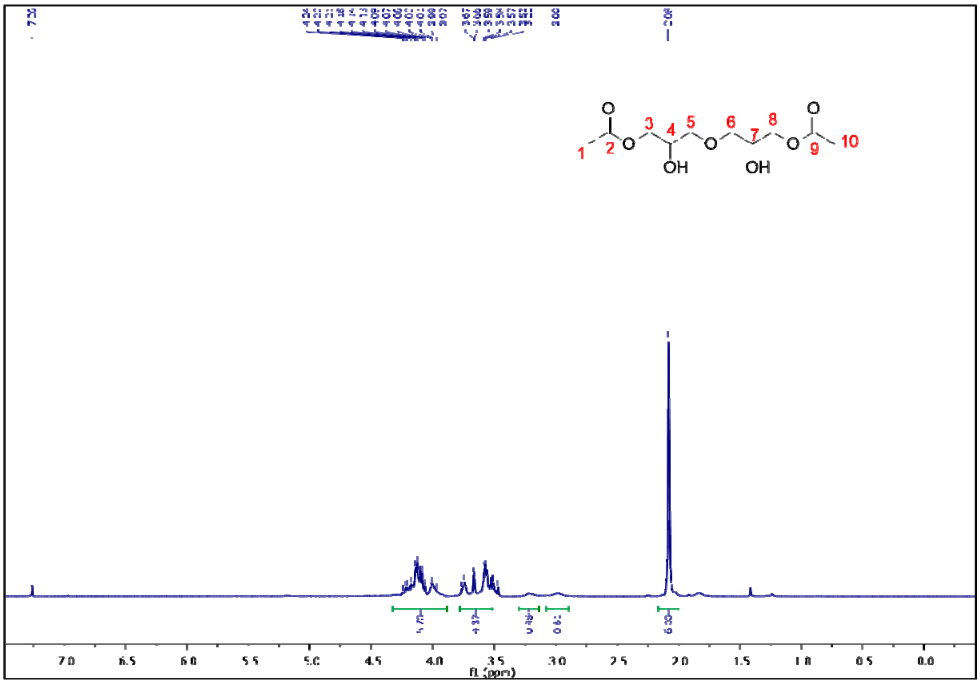

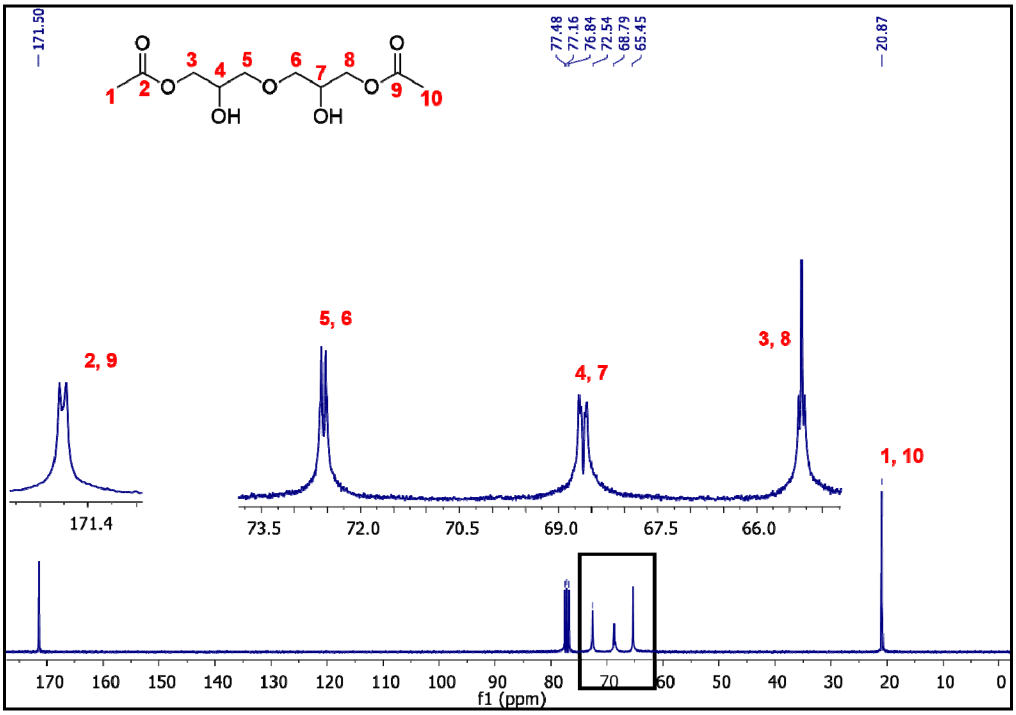

171.50
77.48
77.16
76.84
72.54
68.79
65.45
20.87
2, 9
5, 6
4, 7
3, 8
1, 10
171.4
73.5
72.0
70.5
69.0
67.5
66.0
170 160 150 140 130 120 110 100 90 80 70 60 50 40 30 20 10 0
f1 (ppm)

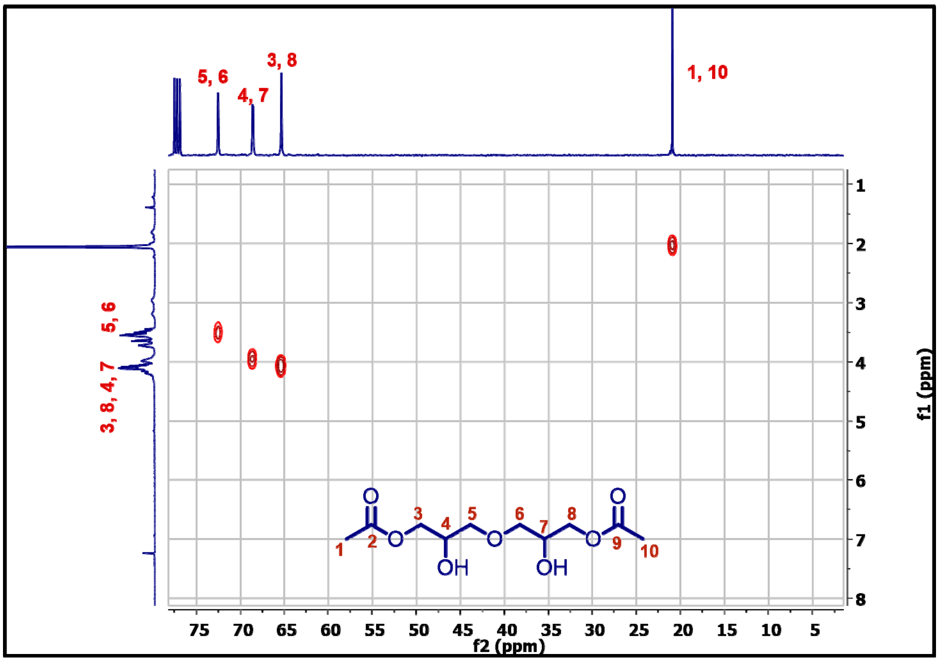

5, 6
4, 7
3, 8
1, 10
5, 6
3, 8, 4, 7
75 70 65 60 55 50 45 40 35 30 25 20 15 10 5
f2 (ppm)
f1 (ppm)

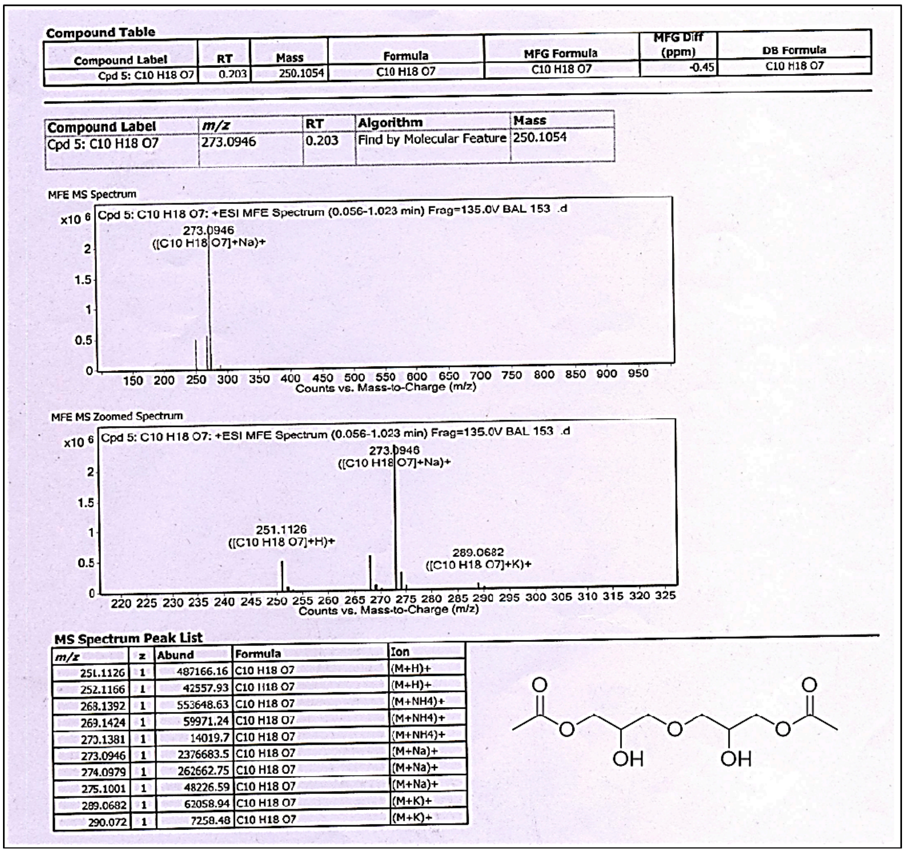

Compound Table

Compound Label	RT	Mass	Formula	MFG Formula	MFG Diff (ppm)	DB Formula
Cpd 5: C10 H18 O7	0.203	250.1054	C10 H18 O7	C10 H18 O7	-0.45	C10 H18 O7

Compound Label	*m/z*	RT	Algorithm	Mass
Cpd 5: C10 H18 O7	273.0946	0.203	Find by Molecular Feature	250.1054

MS Spectrum Peak List

m/z	z	Abund	Formula	Ion
251.1126	1	487166.16	C10 H18 O7	(M+H)+
252.1166	1	42557.93	C10 H18 O7	(M+H)+
268.1392	1	553648.63	C10 H18 O7	(M+NH4)+
269.1424	1	59971.24	C10 H18 O7	(M+NH4)+
270.1381	1	14019.7	C10 H18 O7	(M+NH4)+
273.0946	1	2376683.5	C10 H18 O7	(M+Na)+
274.0979	1	262662.75	C10 H18 O7	(M+Na)+
275.1001	1	48226.59	C10 H18 O7	(M+Na)+
289.0682	1	62058.94	C10 H18 O7	(M+K)+
290.072	1	7258.48	C10 H18 O7	(M+K)+

Figure S1. ^{1}H-, ^{13}C-NMR, HETCORE and HRMS spectra of compound **2**.

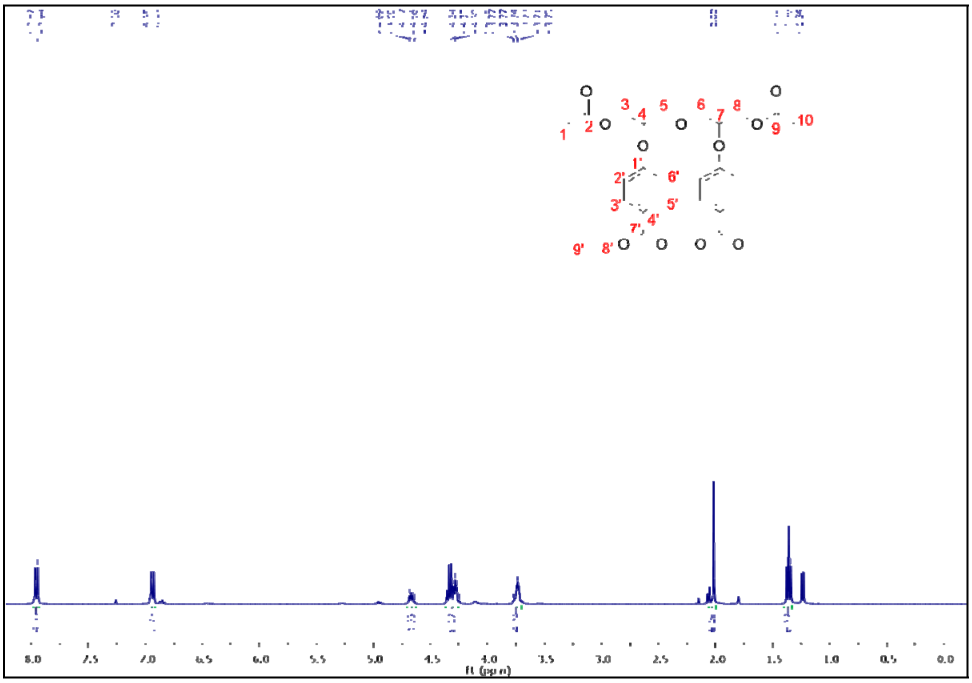

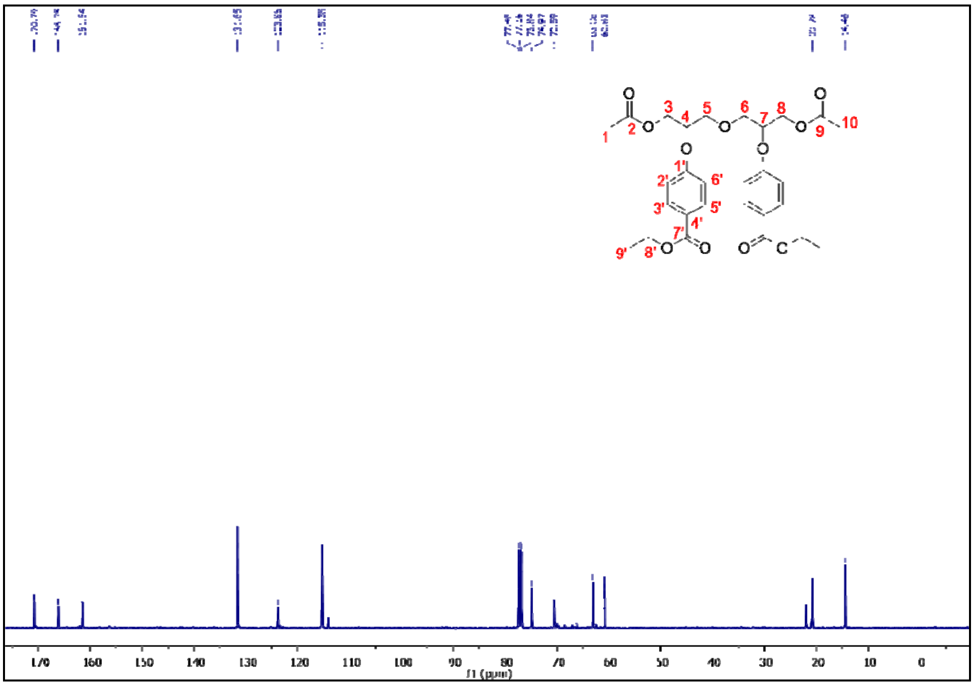

Compound Table

Compound Label	RT	Mass	Formula	MFG Formula	MFG Diff (ppm)	DB Formula
Cpd 1: C28 H34 O11	0.201	546.2088	C28 H34 O11	C28 H34 O11	2.35	C28 H34 O11

Compound Label	*m/z*	RT	Algorithm	Mass
Cpd 1: C28 H34 O11	547.2162	0.201	Find by Molecular Feature	546.2088

MFE MS Spectrum

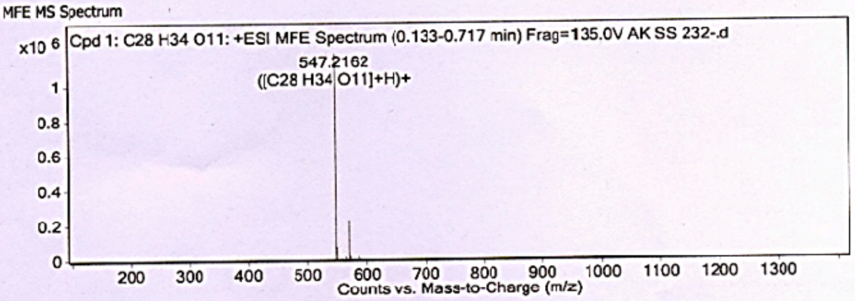

MFE MS Zoomed Spectrum

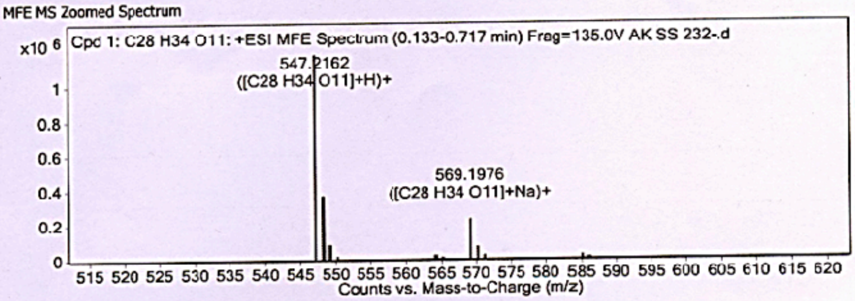

MS Spectrum Peak List

m/z	z	Abund	Formula	Ion
547.2162	1	1180624.25	C28 H34 O11	(M+H)+
548.2194	1	364757.53	C28 H34 O11	(M+H)+
549.2214	1	76058.26	C28 H34 O11	(M+H)+
550.2238	1	12465.6	C28 H34 O11	(M+H)+
564.2417	1	17582.48	C28 H34 O11	(M+NH4)+
565.2449	1	5514.71	C28 H34 O11	(M+NH4)+
569.1976	1	225160.56	C28 H34 O11	(M+Na)+
570.2009	1	67233.68	C28 H34 O11	(M+Na)+
571.2031	1	15374.28	C28 H34 O11	(M+Na)+
585.1713	1	15611.32	C28 H34 O11	(M+K)+

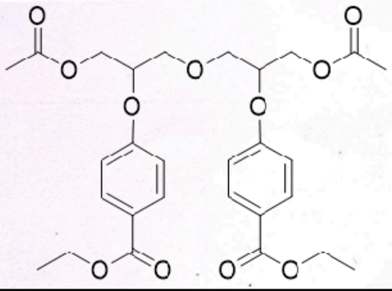

Figure S2. ^{1}H-, ^{13}C-NMR and HRMS spectra of compound **3**.

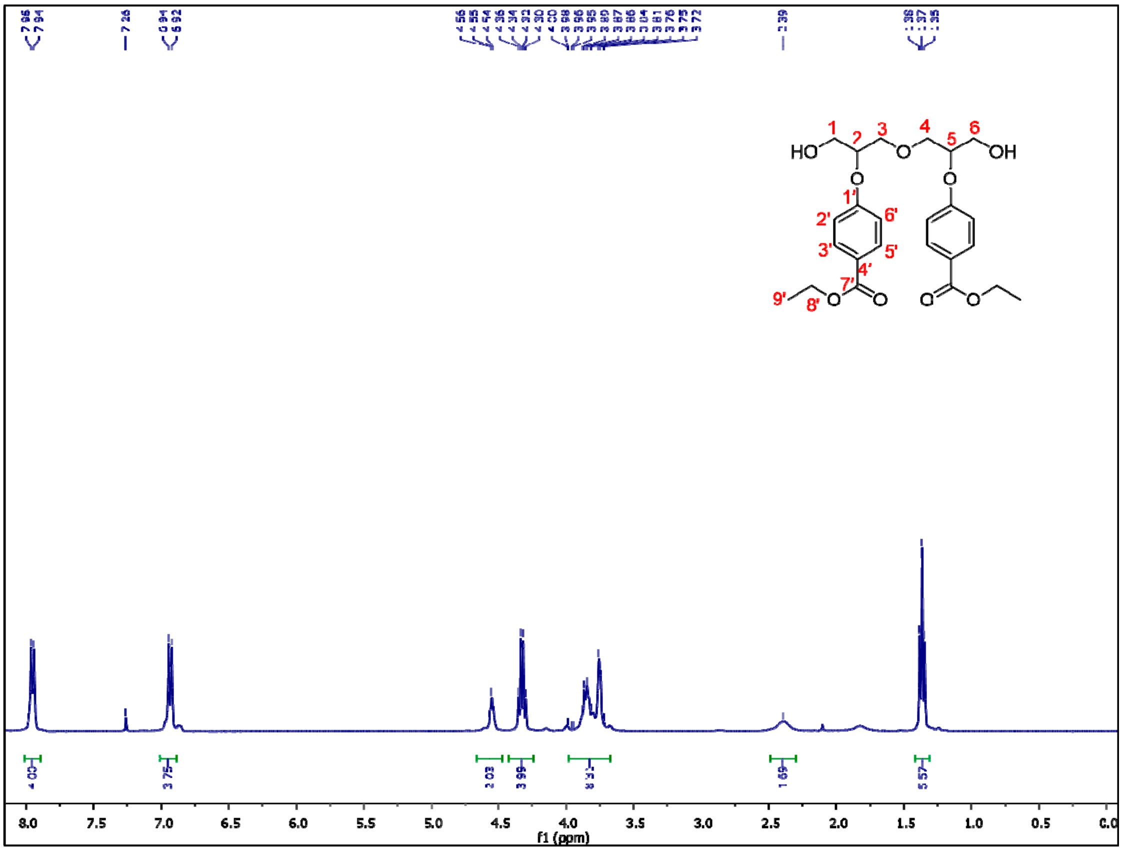
HO
OH
f1 (ppm)

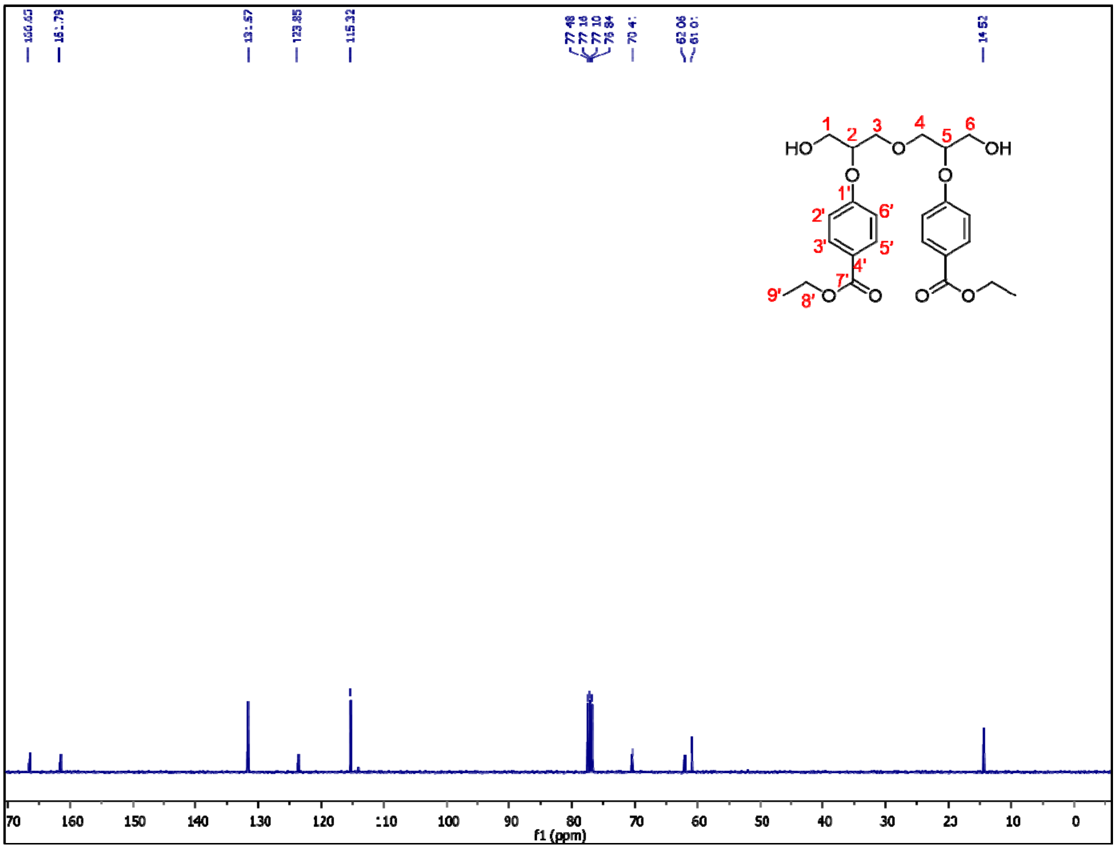
HO
OH
f1 (ppm)

Compound Table

Compound Label	RT	Mass	Formula	MFG Formula	MFG Diff (ppm)	DB Formula
Cpd 5: C24 H30 O9	0.202	462.189	C24 H30 O9	C24 H30 O9	-0.06	C24 H30 O9

Compound Label	*m/z*	RT	Algorithm	Mass
Cpd 5: C24 H30 O9	463.1964	0.202	Find by Molecular Feature	462.189

MFE MS Spectrum

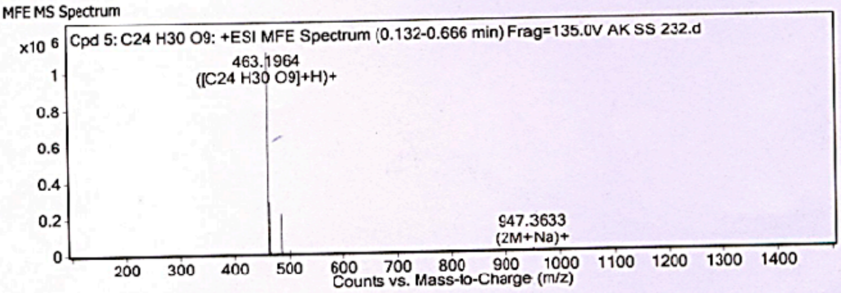

MFE MS Zoomed Spectrum

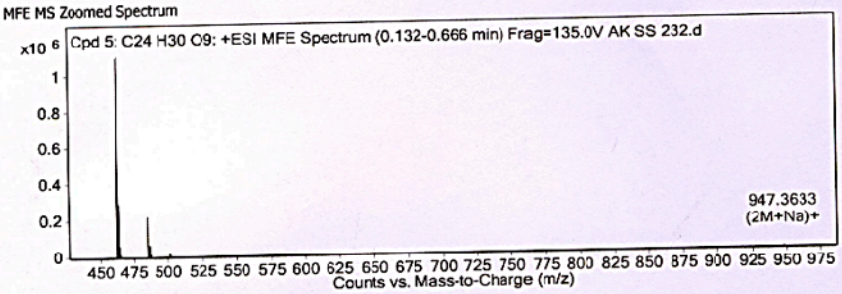

MS Spectrum Peak List

m/z	z	Abund	Formula	Ion
463.1964	1	1105493.88	C24 H30 O9	(M+H)+
464.1996	1	281633.74	C24 H30 O9	(M+H)+
465.2016	1	54743.1	C24 H30 O9	(M+H)+
466.2046	1	8716.68	C24 H30 O9	(M+H)+
485.178	1	217859.14	C24 H30 O9	(M+Na)+
486.1814	1	55751.49	C24 H30 O9	(M+Na)+
487.1729	1	12168.6	C24 H30 O9	(M+Na)+
501.1531	1	15701.36	C24 H30 O9	(M+K)+
502.1583	1	4514.32	C24 H30 O9	(M+K)+
947.3633	1	5901.47		(2M+Na)+

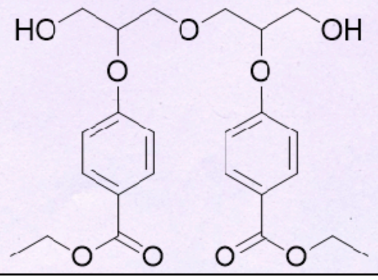

Figure S3. ^{1}H-, ^{13}C-NMR and HRMS spectra of compound **4**.

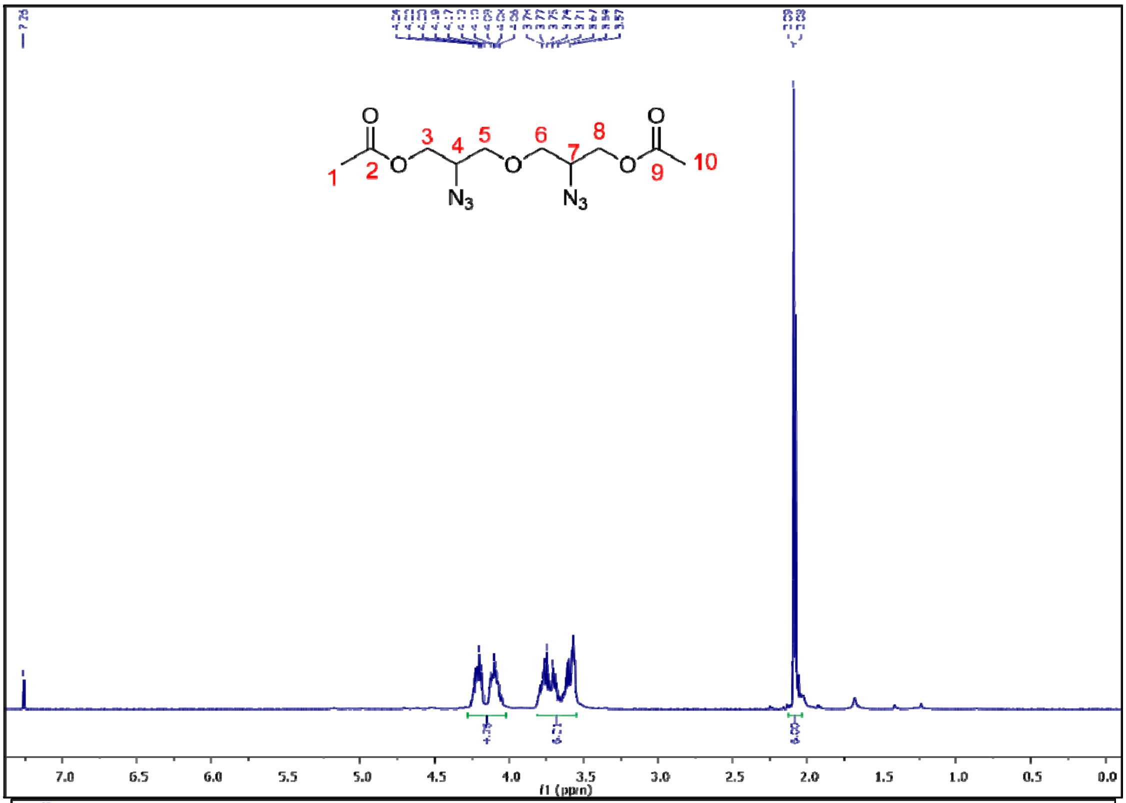
1
2
3
4
5
6
7
8
9
10
O
O
O
O
O
N_3
N_3
f1 (ppm)

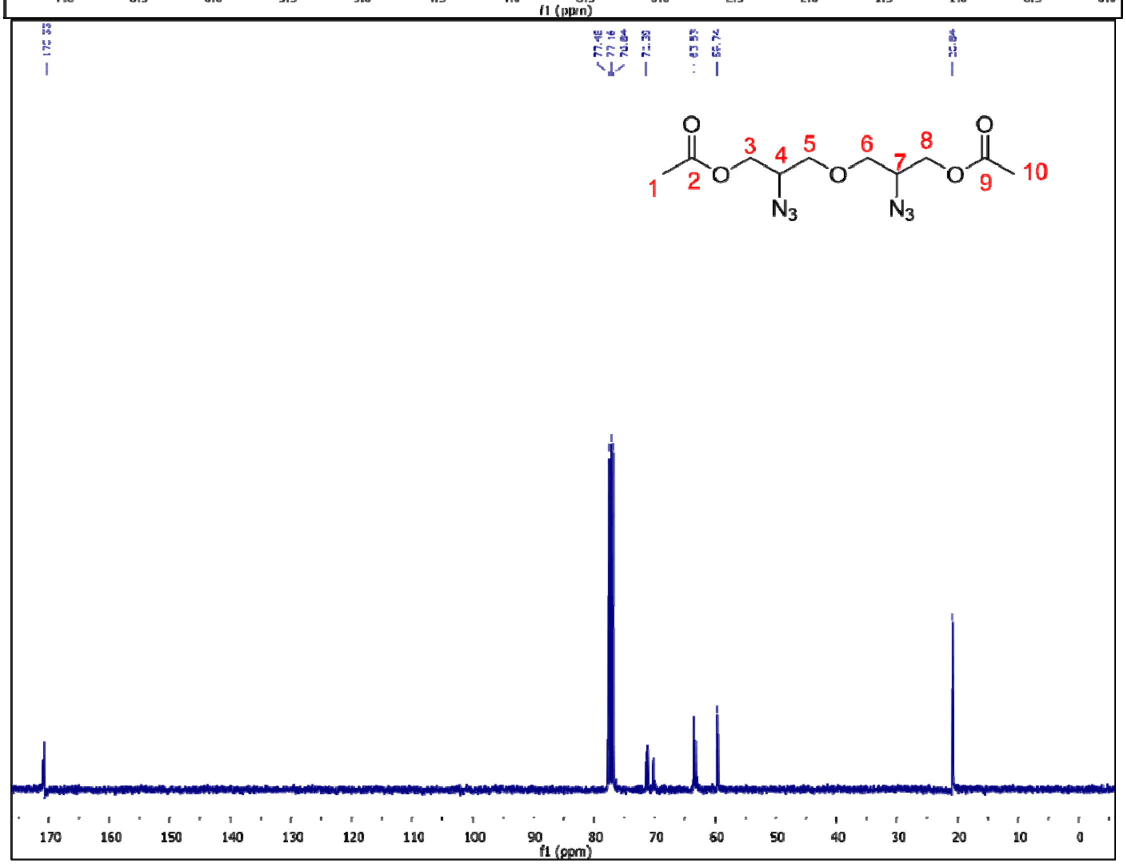
1
2
3
4
5
6
7
8
9
10
O
O
O
O
O
N_3
N_3
f1 (ppm)

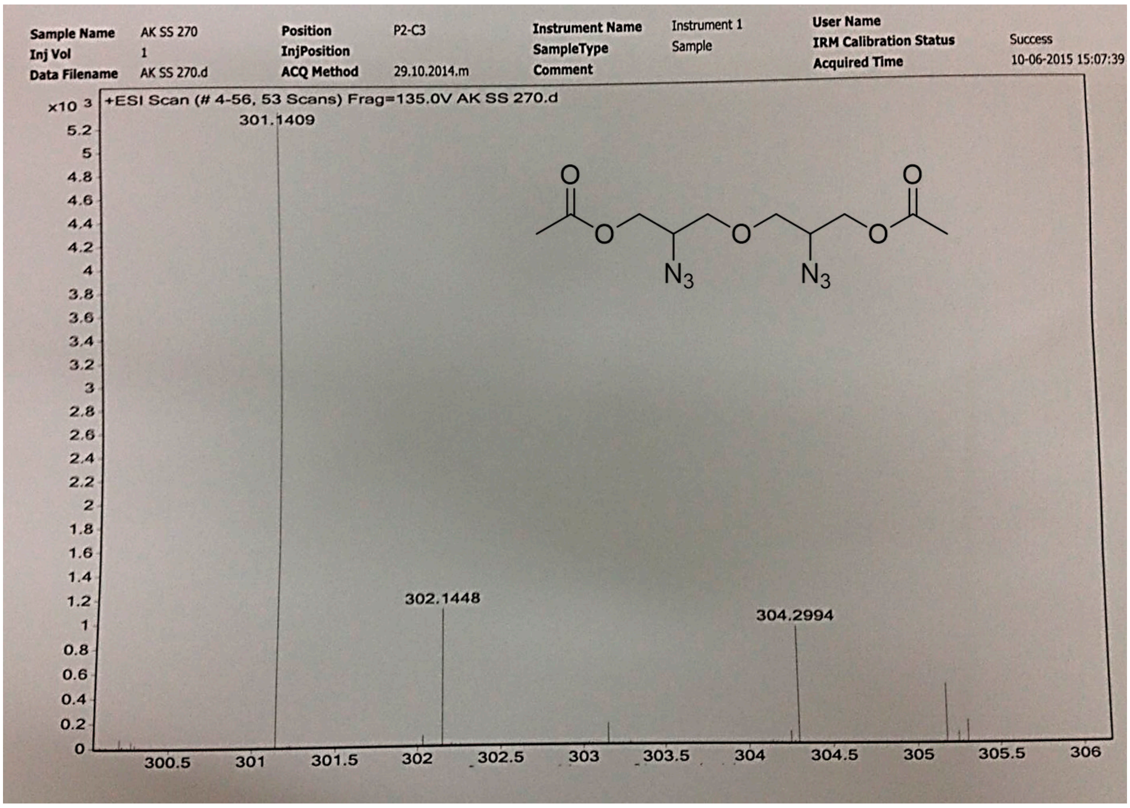

Figure S4. ^{1}H-, ^{13}C-NMR and HRMS spectra of compound **5**.

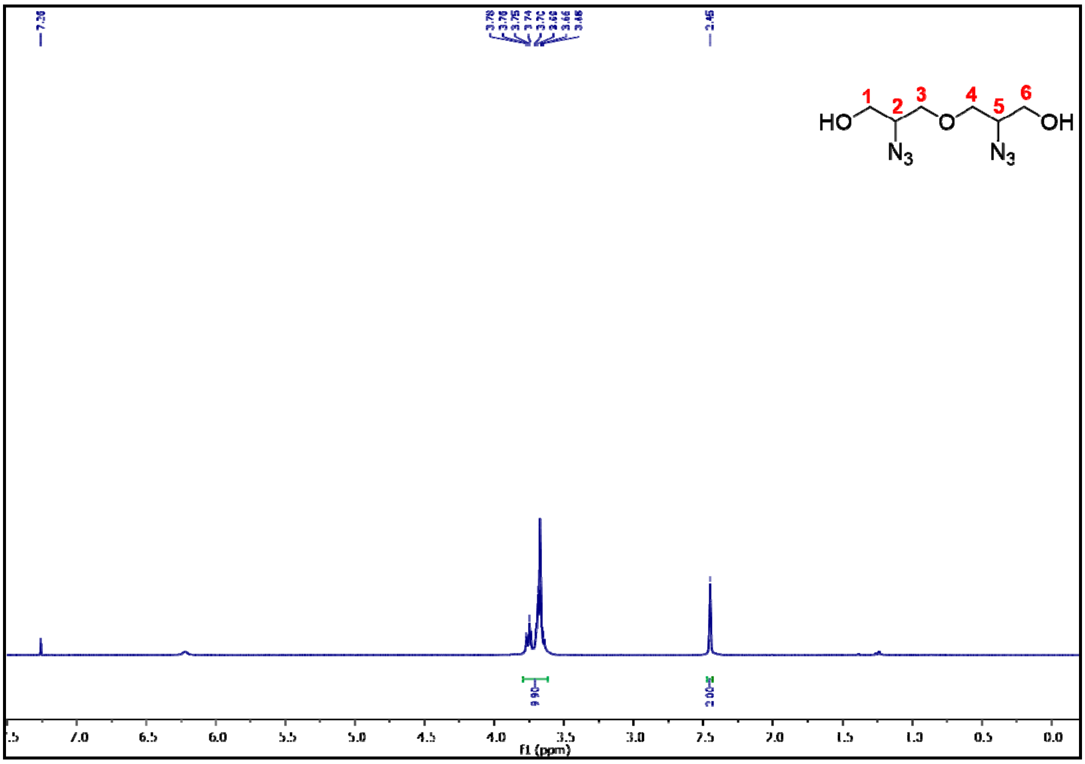

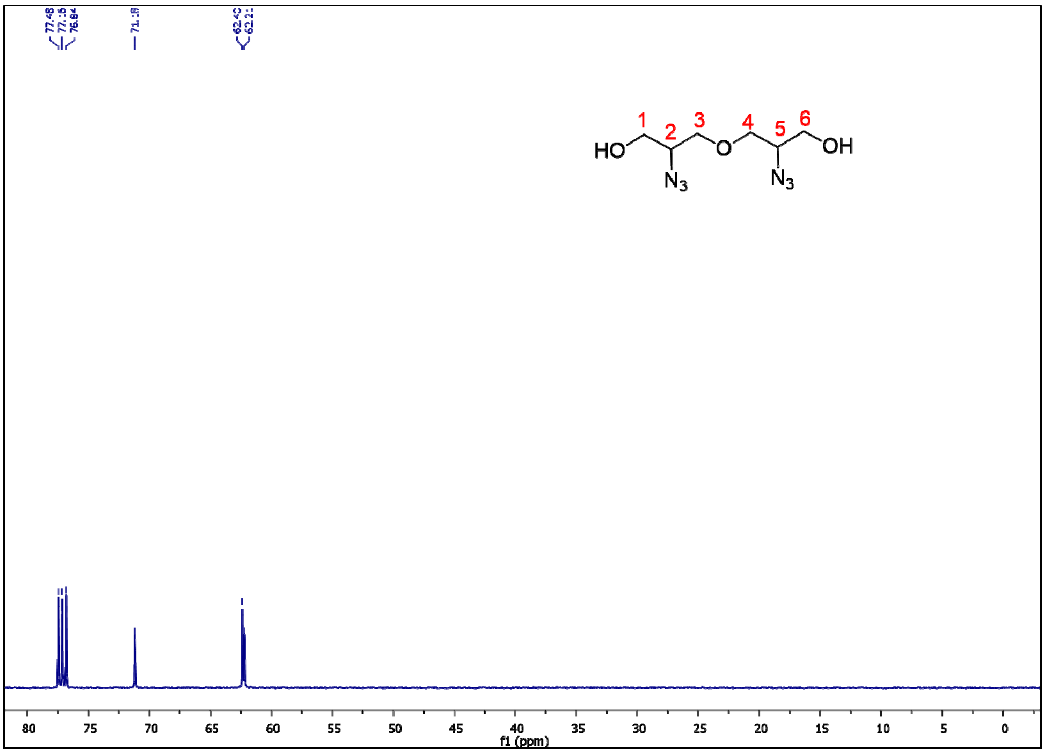

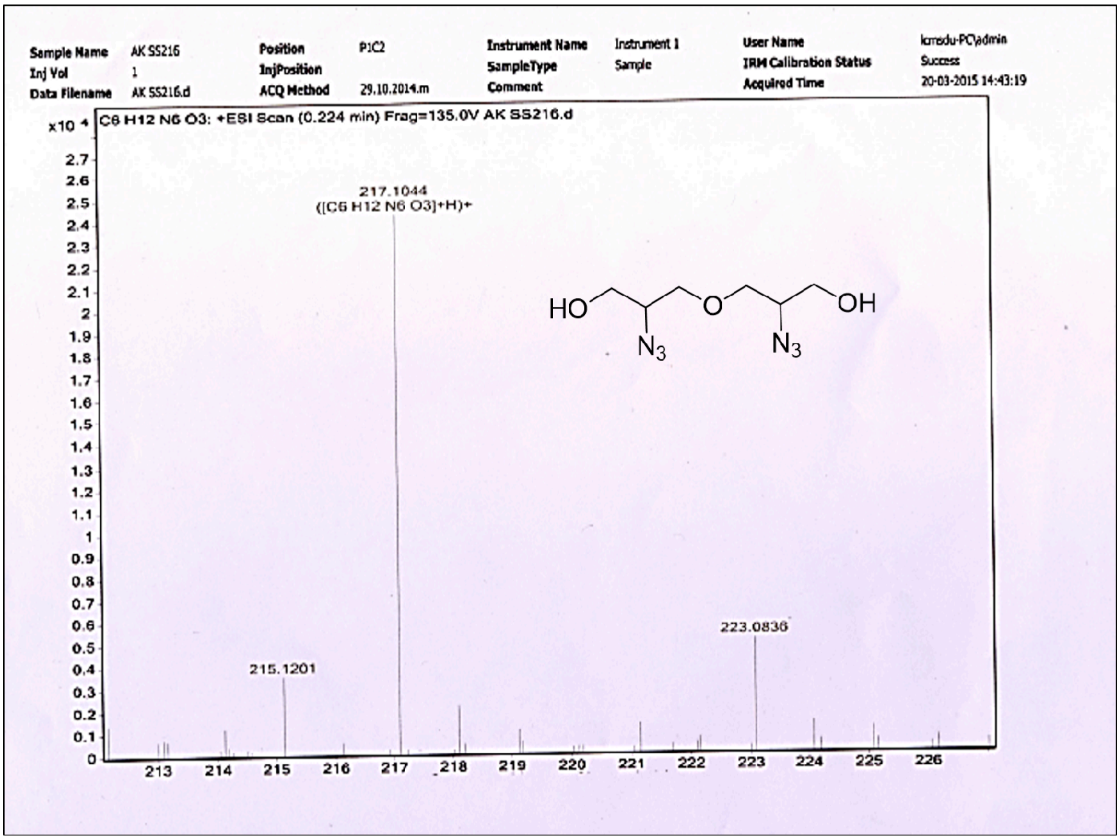

Figure S5. ^{1}H-, ^{13}C-NMR and HRMS spectra of compound **6**.

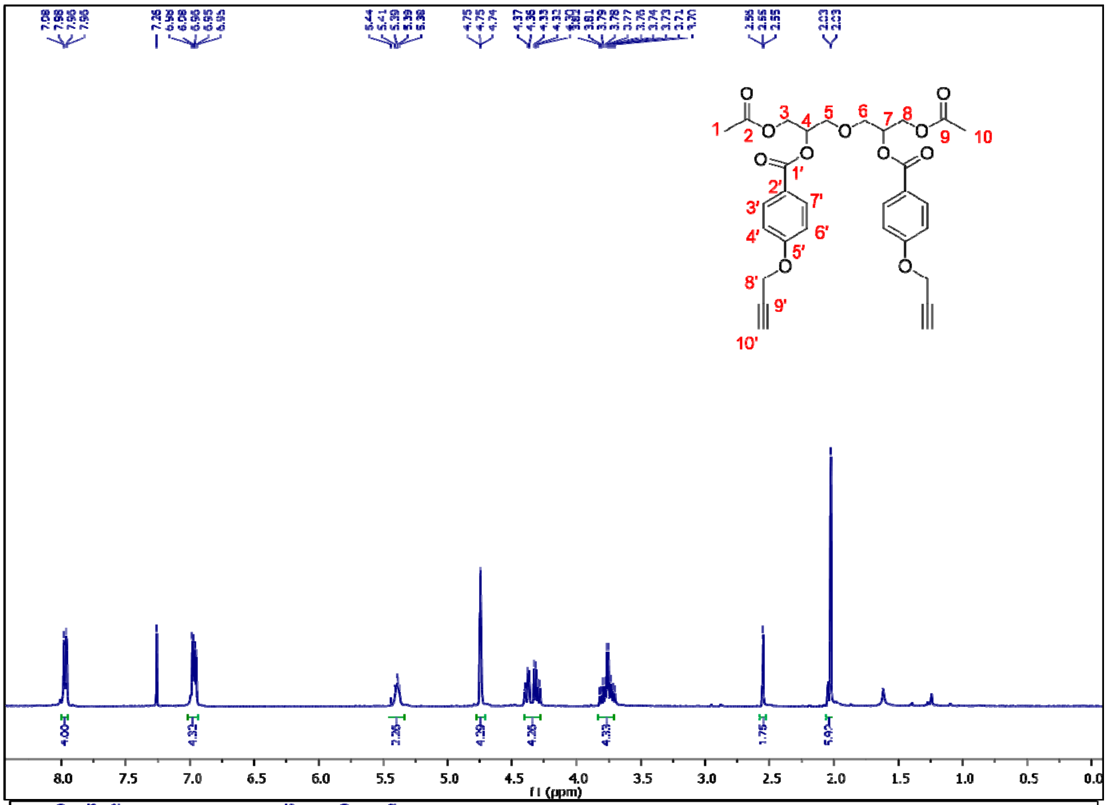

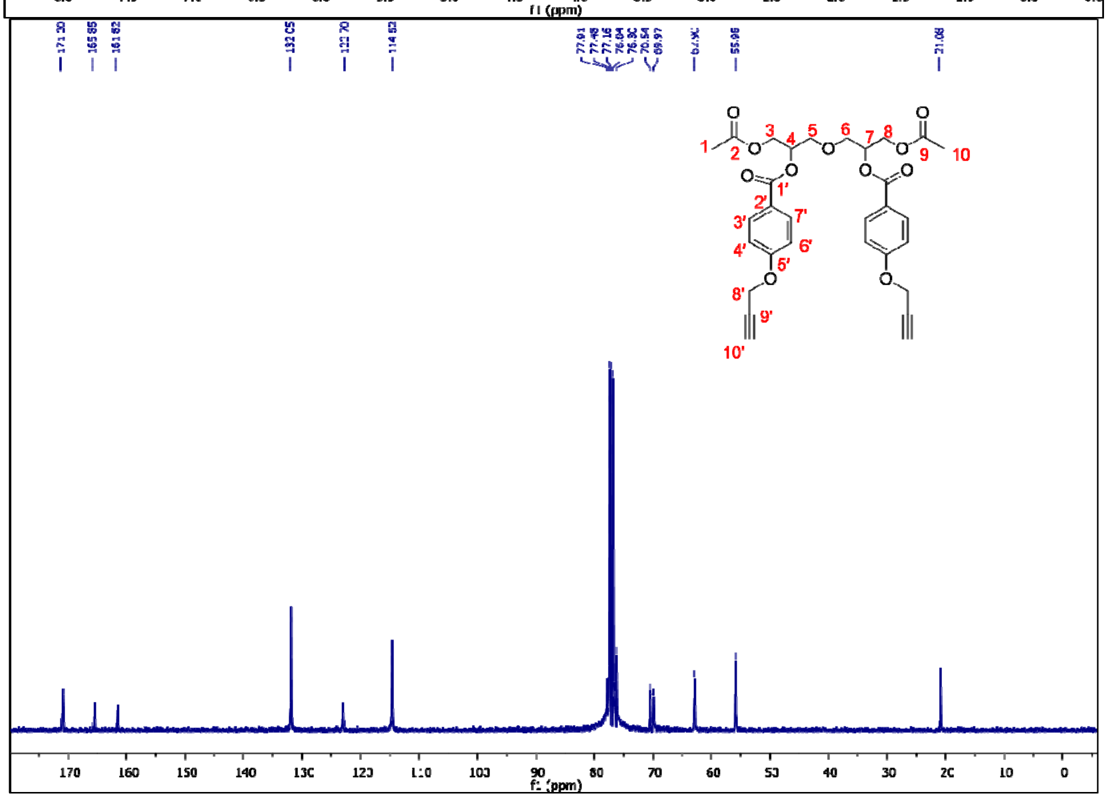

Compound Table

Compound Label	RT	Mass	Formula	MFG Formula	MFG Diff (ppm)	DB Formula
Cpd 1: C30 H30 O11	0.2	566.1783	C30 H30 O11	C30 H30 O11	0.9	C30 H30 O11

Compound Label	m/z	RT	Algorithm	Mass
Cpd 1: C30 H30 O11	584.2123	0.2	Find by Molecular Feature	566.1783

MFE MS Spectrum

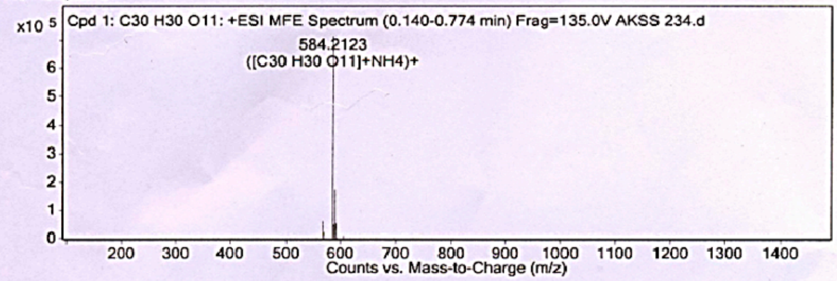

MFE MS Zoomed Spectrum

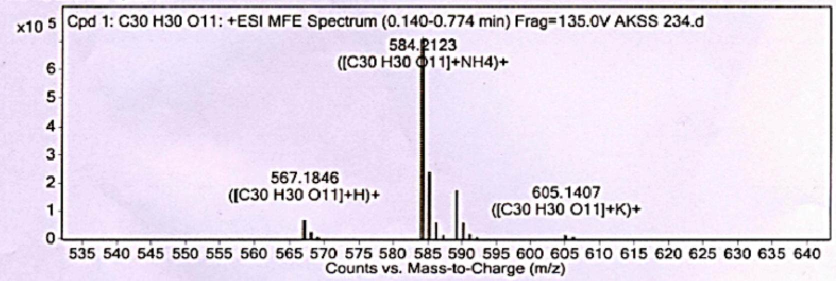

MS Spectrum Peak List

m/z	z	Abund	Formula	Ion
567.1846	1	61419.93	C30 H30 O11	(M+H)+
568.1877	1	21607.85	C30 H30 O11	(M+H)+
584.2123	1	715536	C30 H30 O11	(M+NH4)+
585.2153	1	228638.63	C30 H30 O11	(M+NH4)+
586.2173	1	49929.41	C30 H30 O11	(M+NH4)+
587.219	1	9039.81	C30 H30 O11	(M+NH4)+
589.1669	1	172121.38	C30 H30 O11	(M+Na)+
590.1703	1	55282.26	C30 H30 O11	(M+Na)+
591.1732	1	12283.34	C30 H30 O11	(M+Na)+
605.1407	1	9660.31	C30 H30 O11	(M+K)+

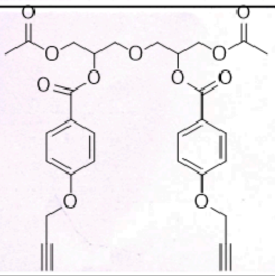

Figure S6. ^{1}H-, ^{13}C-NMR and HRMS spectra of compound **7**.

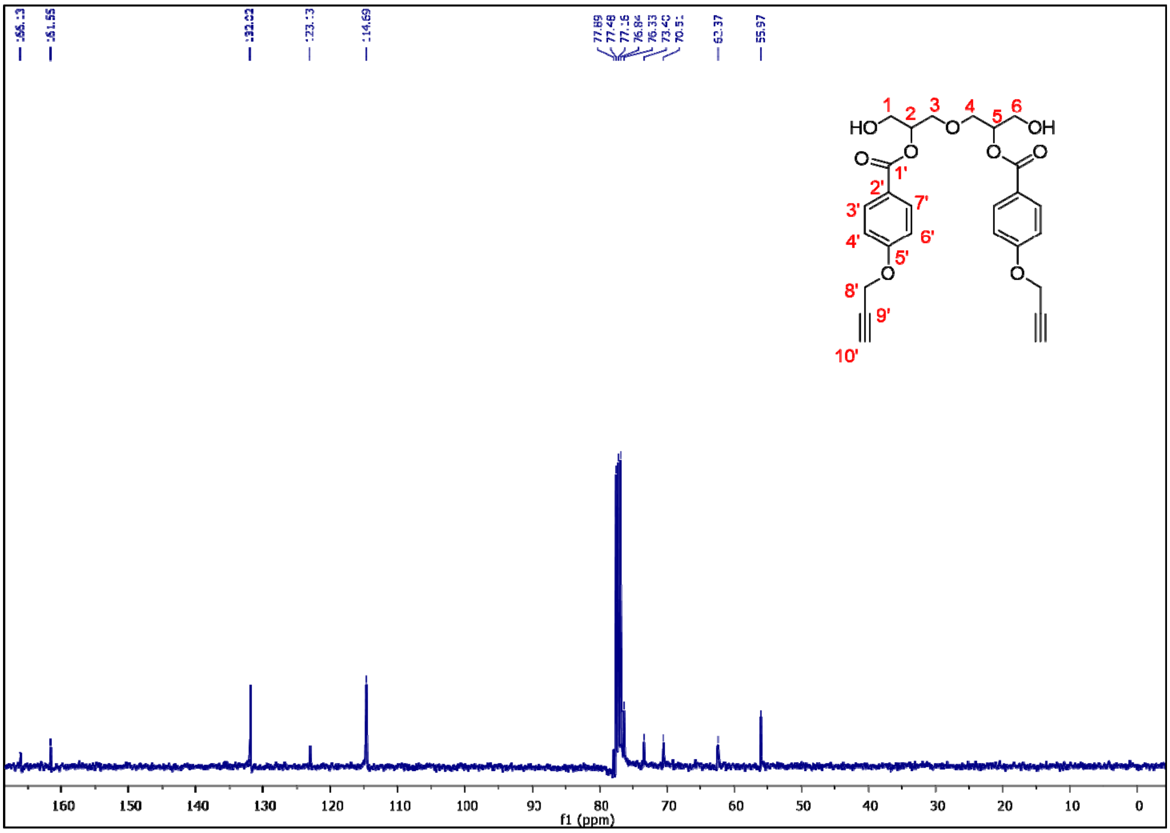

f1 (ppm)
HO
OH
1
2
3
4
5
6
1'
2'
3'
4'
5'
6'
7'
8'
9'
10'

Compound Table

Compound Label	RT	Mass	Formula	MFG Formula	MFG Diff (ppm)	DB Formula
Cpd 2: C26 H26 O9	0.2	482.1566	C26 H26 O9	C26 H26 O9	2.15	C26 H26 O9

Compound Label	*m/z*	RT	Algorithm	Mass
Cpd 2: C26 H26 O9	505.1459	0.2	Find by Molecular Feature	482.1566

MFE MS Spectrum

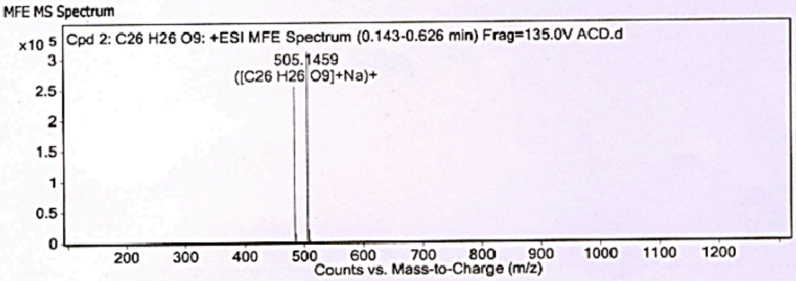

MFE MS Zoomed Spectrum

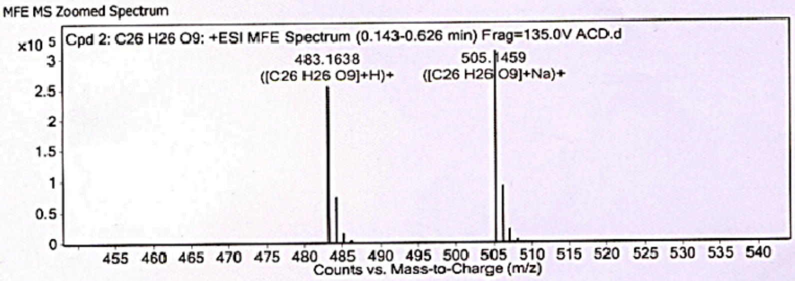

MS Spectrum Peak List

m/z	z	Abund	Formula	Ion
483.1638	1	254020.02	C26 H26 O9	(M+H)+
484.1671	1	70416.36	C26 H26 O9	(M+H)+
485.1695	1	14388.49	C26 H26 O9	(M+H)+
486.1703	1	2408.07	C26 H26 O9	(M+H)+
487.1761	1	122.94	C26 H26 O9	(M+H)+
505.1459	1	310696.19	C26 H26 O9	(M+Na)+
506.1492	1	87666.68	C26 H26 O9	(M+Na)+
507.1523	1	20719.18	C26 H26 O9	(M+Na)+
508.1545	1	3685.04	C26 H26 O9	(M+Na)+
509.1488	1	829.18	C26 H26 O9	(M+Na)+

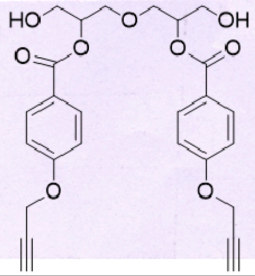

Figure S7. ^{1}H-, ^{13}C-NMR and HRMS spectra of compound **8**.

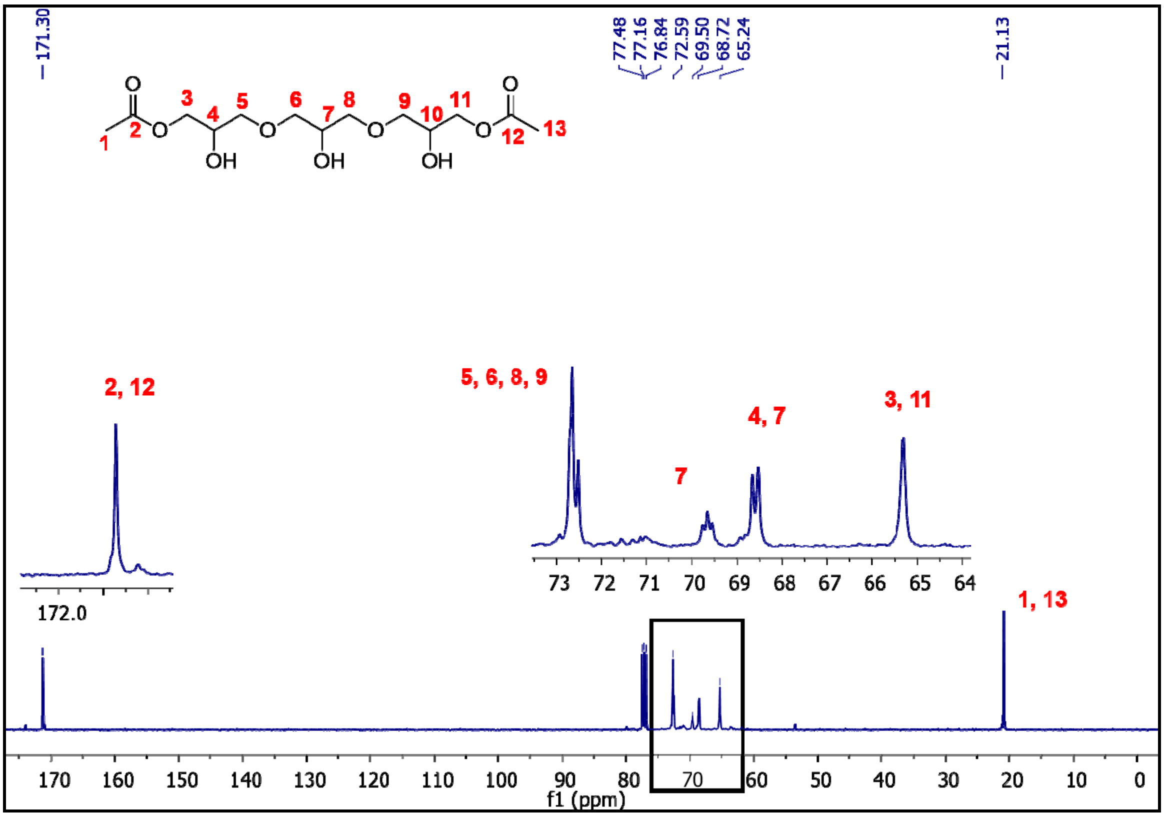
171.30
77.48
77.16
76.84
72.59
69.50
68.72
65.24
21.13
1
2
3
4
5
6
7
8
9
10
11
12
13
O
OH
2, 12
172.0
5, 6, 8, 9
7
4, 7
3, 11
73 72 71 70 69 68 67 66 65 64
1, 13
170 160 150 140 130 120 110 100 90 80 70 60 50 40 30 20 10 0
f1 (ppm)

Compound Table

Compound Label	RT	Mass	Formula	MFG Formula	MFG Diff (ppm)	DB Formula
Cpd 5: C13 H24 O9	0.204	324.1416	C13 H24 O9	C13 H24 O9	1.24	C13 H24 O9

Compound Label	*m/z*	RT	Algorithm	Mass
Cpd 5: C13 H24 O9	347.1309	0.204	Find by Molecular Feature	324.1416

MFE MS Spectrum

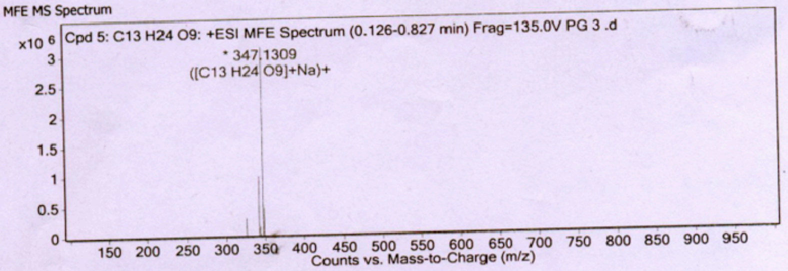

MFE MS Zoomed Spectrum

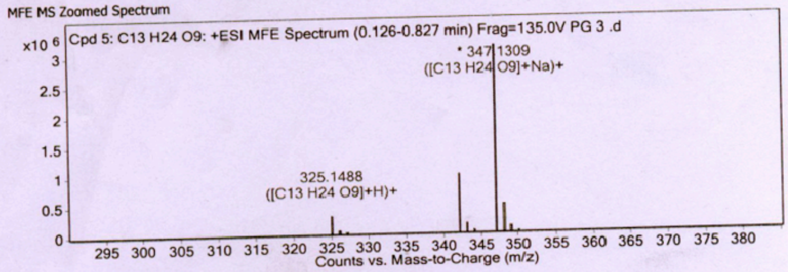

MS Spectrum Peak List

m/z	z	Abund	Formula	Ion
325.1488	1	288270.88	C13 H24 O9	(M+H)+
326.1521	1	41068.72	C13 H24 O9	(M+H)+
327.1536	1	8488.43	C13 H24 O9	(M+H)+
342.1759	1	984878.63	C13 H24 O9	(M+NH4)+
343.1786	1	145896.22	C13 H24 O9	(M+NH4)+
344.1745	1	34272.38	C13 H24 O9	(M+NH4)+
347.1309	1	3130392	C13 H24 O9	(M+Na)+
348.1342	1	467536.4	C13 H24 O9	(M+Na)+
349.1356	1	94668.31	C13 H24 O9	(M+Na)+
350.1382	1	11114.19	C13 H24 O9	(M+Na)+

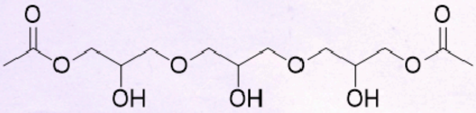

Figure S8. ^{1}H-, ^{13}C-NMR and HRMS spectra of compound **10**.

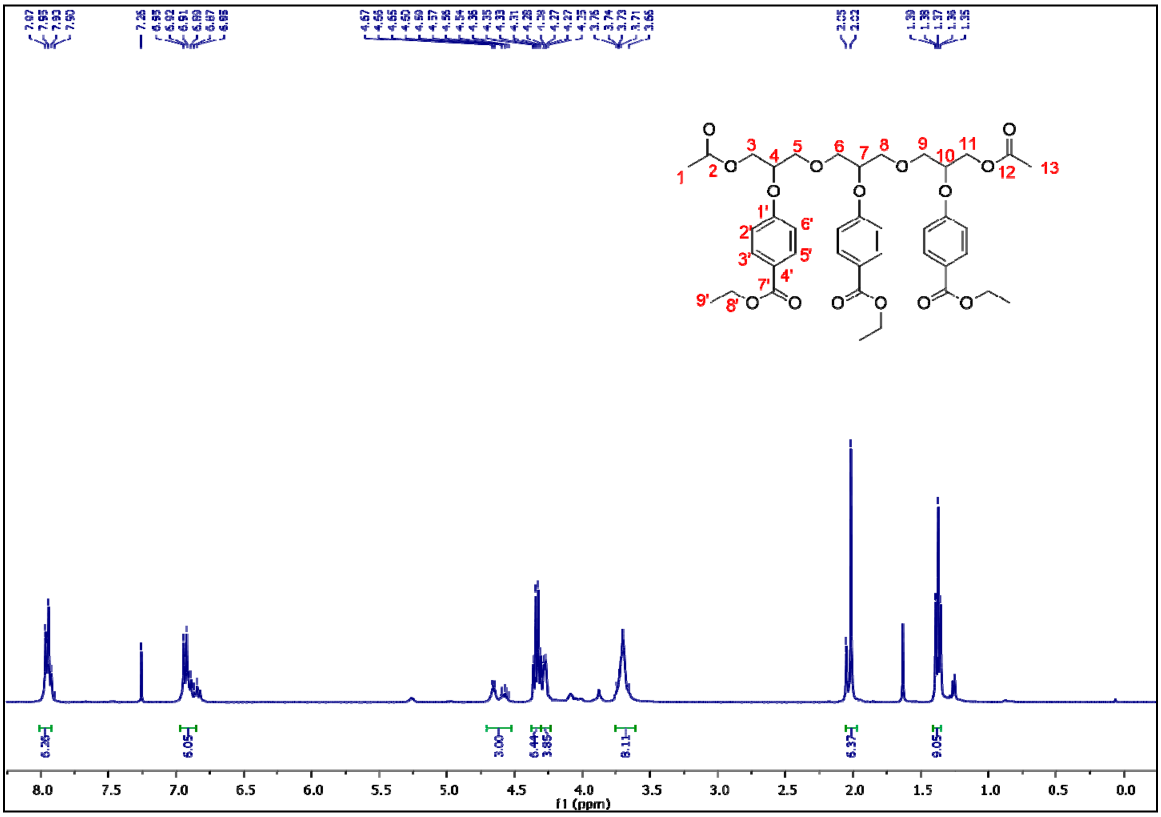

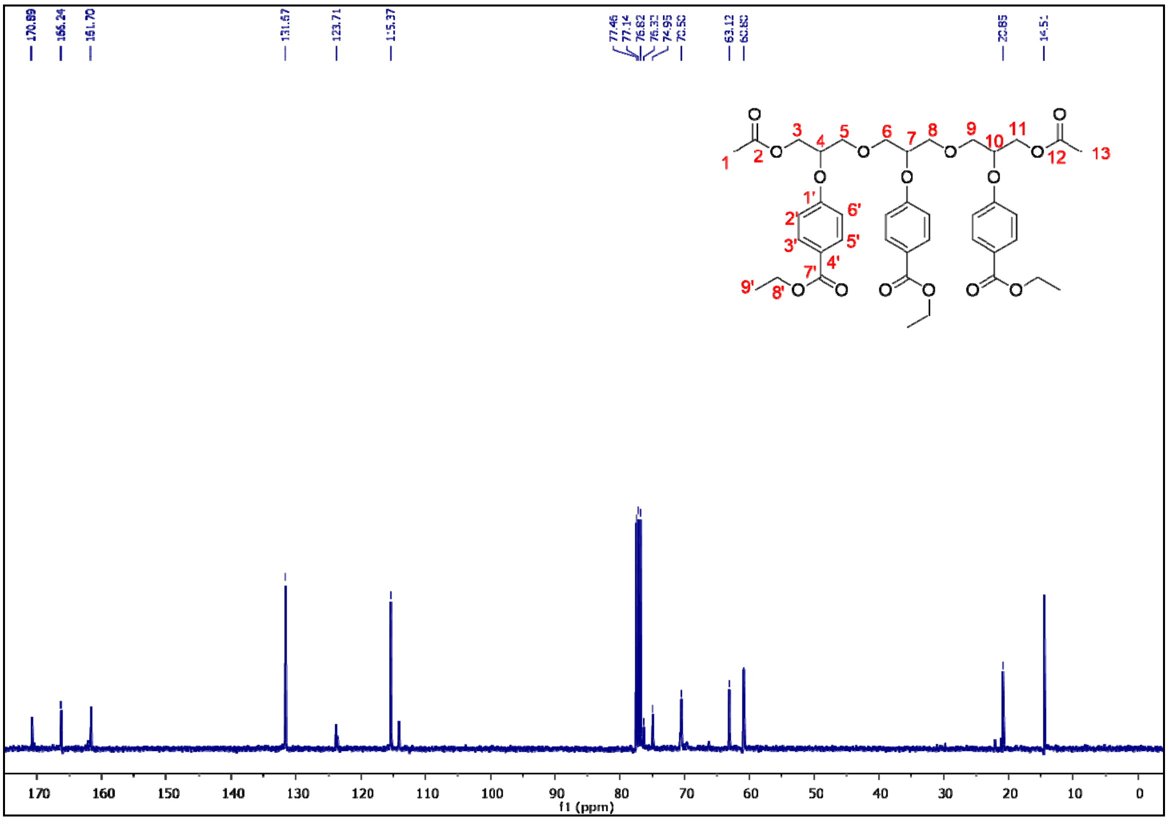

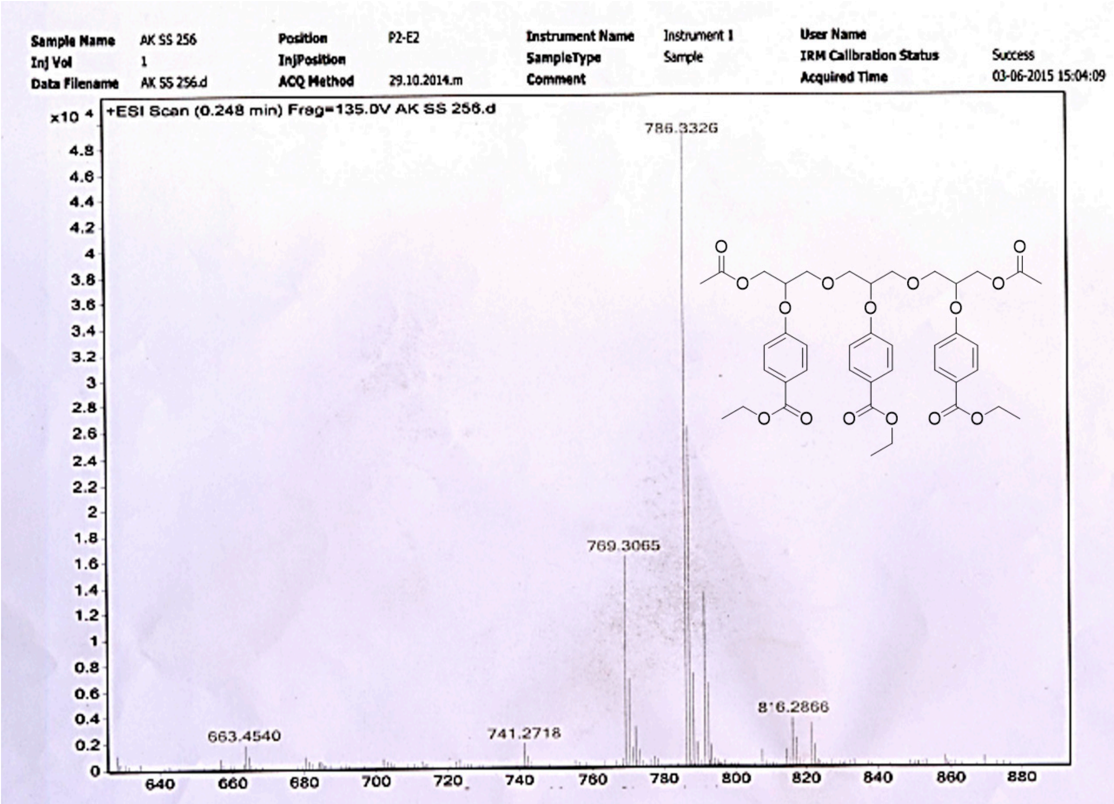

Figure S9. ^{1}H-, ^{13}C-NMR and HRMS spectra of compound **11**.

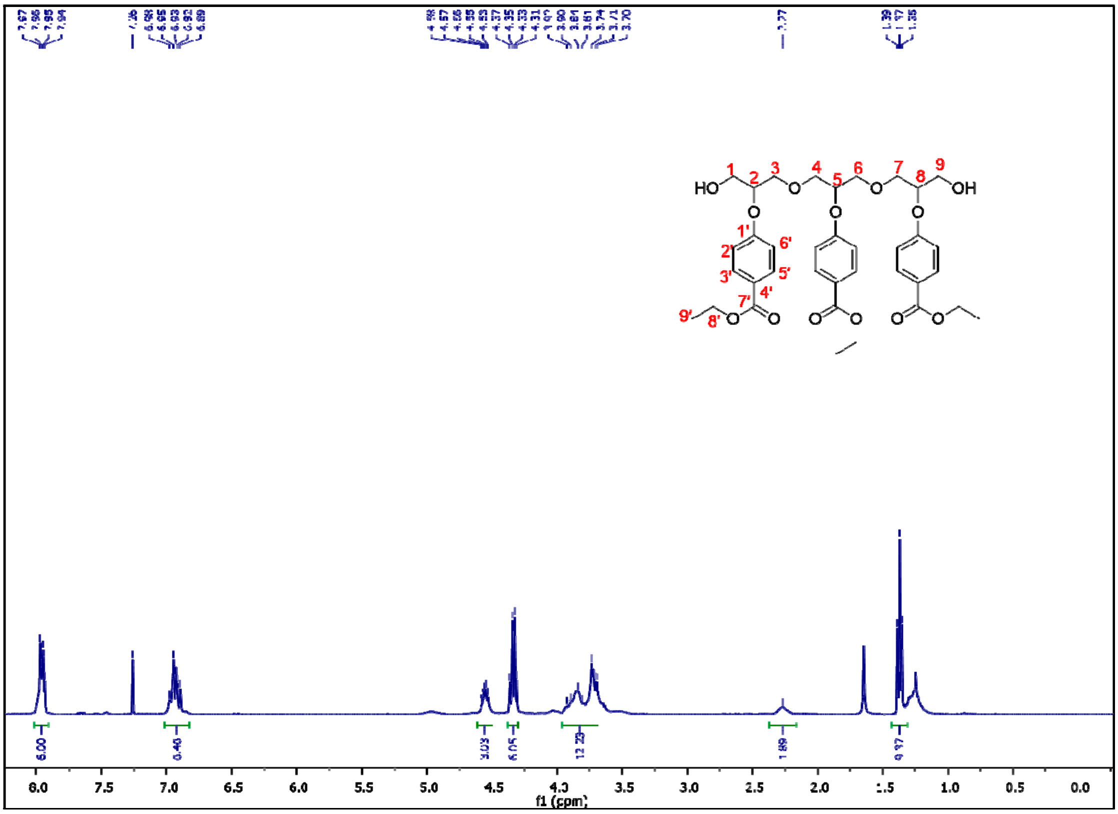

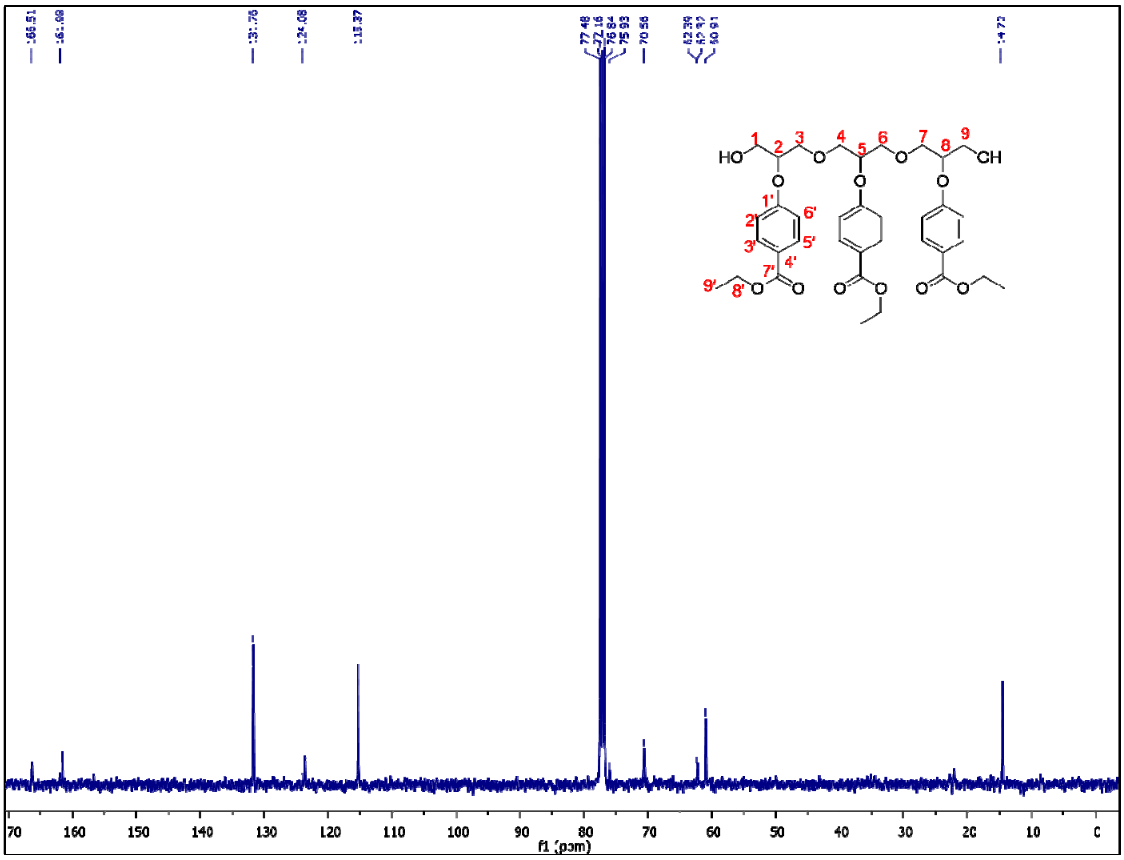

Figure S10. ^{1}H-, ^{13}C-NMR and HRMS spectra of compound **12**.

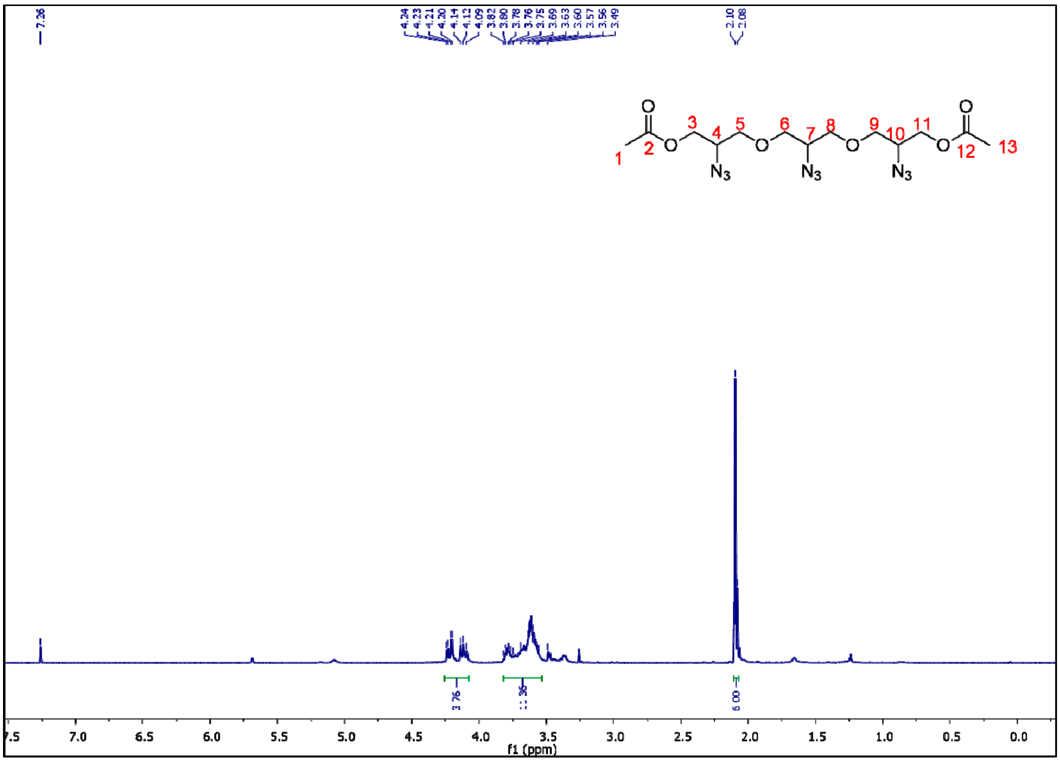

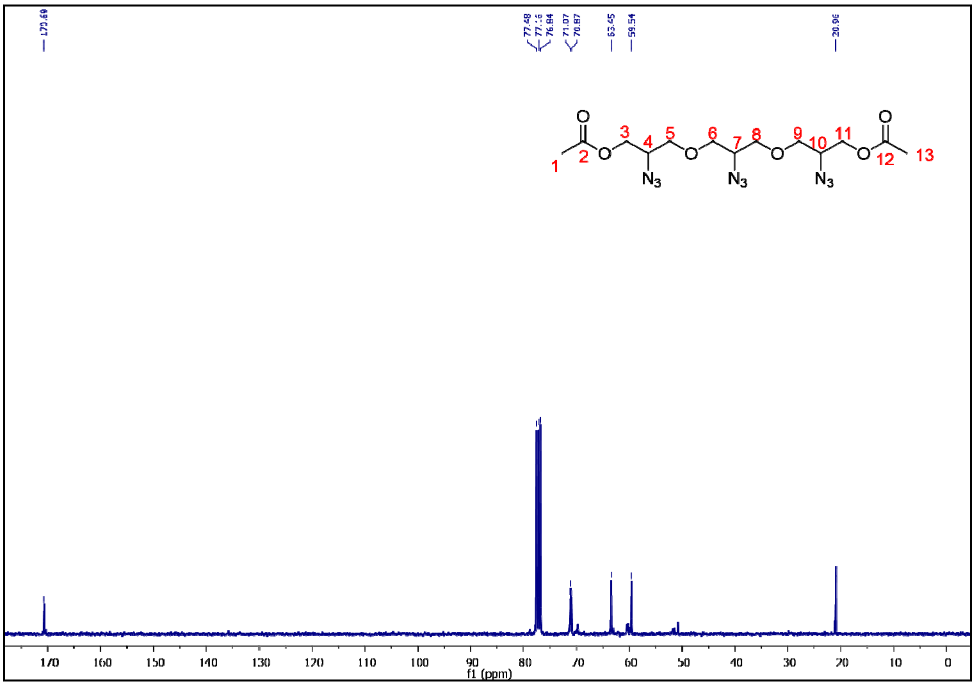

Compound Table

Compound Label	RT	Mass	Formula	MFG Formula	MFG Diff (ppm)	DB Formula
Cpd 4: C13 H21 N9 O6	0.205	399.1633	C13 H21 N9 O6	C13 H21 N9 O6	-4.59	C13 H21 N9 O6

Compound Label	*m/z*	RT	Algorithm	Mass
Cpd 4: C13 H21 N9 O6	417.1971	0.205	Find by Molecular Feature	399.1633

MFE MS Spectrum

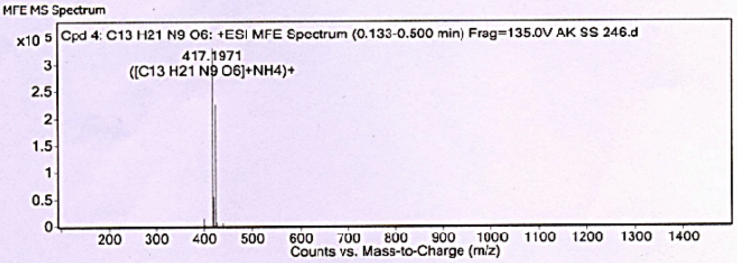

MFE MS Zoomed Spectrum

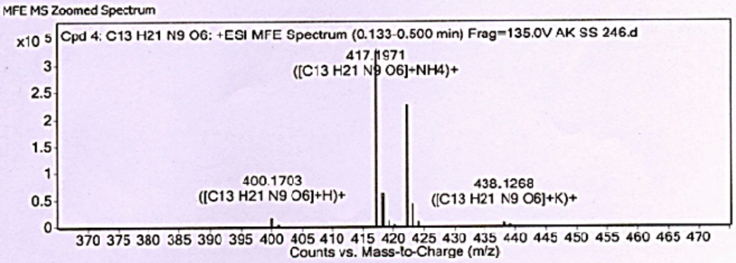

MS Spectrum Peak List

m/z	z	Abund	Formula	Ion
400.1703	1	12886.65	C13 H21 N9 O6	(M+H)+
401.1734	1	2369.79	C13 H21 N9 O6	(M+H)+
417.1971	1	330223.28	C13 H21 N9 O6	(M+NH4)+
418.1993	1	54576.41	C13 H21 N9 O6	(M+NH4)+
419.2017	1	8491.02	C13 H21 N9 O6	(M+NH4)+
420.2041	1	1122.91	C13 H21 N9 O6	(M+NH4)+
422.1526	1	227093.55	C13 H21 N9 O6	(M+Na)+
423.1547	1	38404.7	C13 H21 N9 O6	(M+Na)+
424.1569	1	6158.59	C13 H21 N9 O6	(M+Na)+
438.1268	1	4950.77	C13 H21 N9 O6	(M+K)+

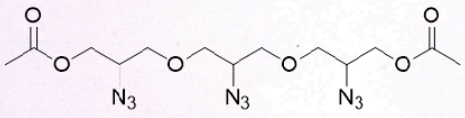

Figure S11. ^{1}H, ^{13}C-NMR and HRMS spectra of compound **13**.

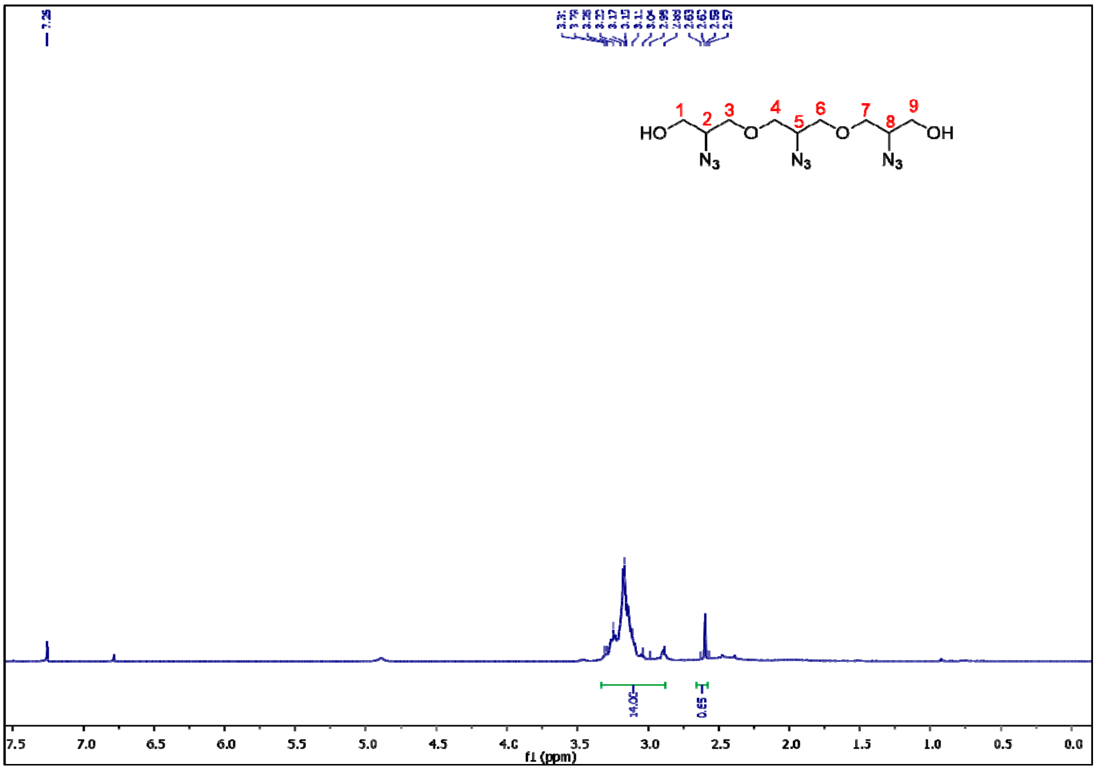

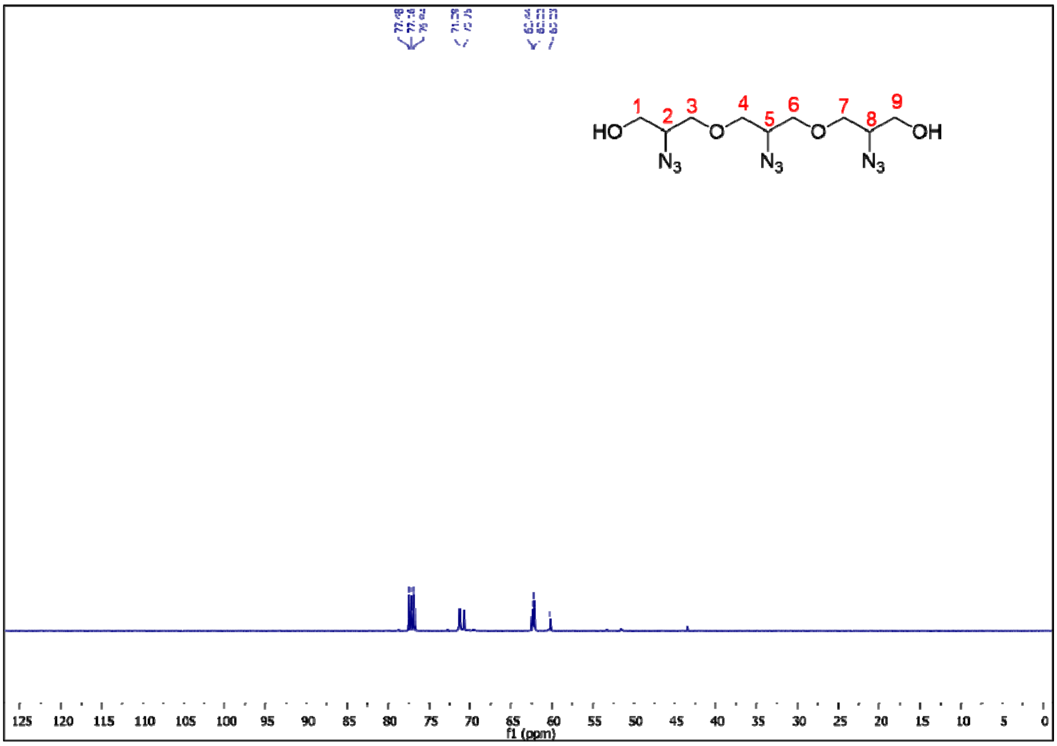

Compound Table

Compound Label	RT	Mass	Formula	MFG Formula	MFG Diff (ppm)	DB Formula
Cpd 5: C9 H17 N9 O4	0.209	315.1405	C9 H17 N9 O4	C9 H17 N9 O4	-0.61	C9 H17 N9 O4

Compound Label	m/z	RT	Algorithm	Mass
Cpd 5: C9 H17 N9 O4	338.1298	0.209	Find by Molecular Feature	315.1405

MFE MS Spectrum

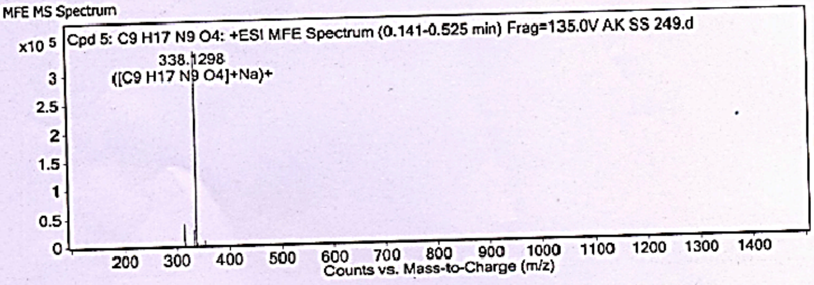

MFE MS Zoomed Spectrum

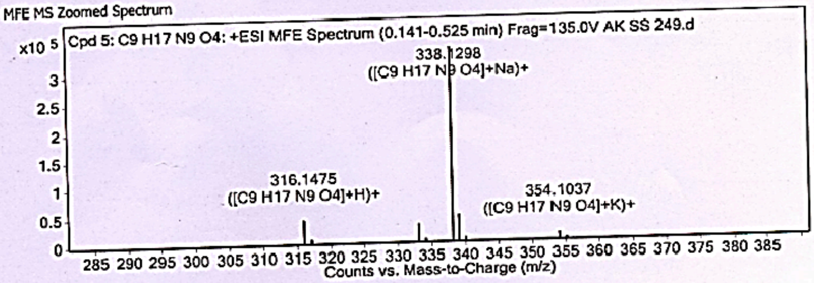

MS Spectrum Peak List

m/z	z	Abund	Formula	Ion
316.1475	1	39392.68	C9 H17 N9 O4	(M+H)+
317.1481	1	5469.77	C9 H17 N9 O4	(M+H)+
333.1742	1	29034.58	C9 H17 N9 O4	(M+NH4)+
334.176	1	4002.84	C9 H17 N9 O4	(M+NH4)+
338.1298	1	342203.06	C9 H17 N9 O4	(M+Na)+
339.1315	1	44444.12	C9 H17 N9 O4	(M+Na)+
340.1308	1	5858.99	C9 H17 N9 O4	(M+Na)+
354.1037	1	9550.18	C9 H17 N9 O4	(M+K)+
355.0976	1	1466.58	C9 H17 N9 O4	(M+K)+
356.0984	1	990.74	C9 H17 N9 O4	(M+K)+

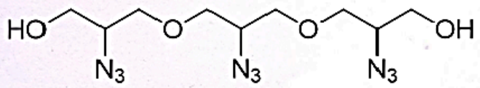

Figure S12. ^{1}H-, ^{13}C-NMR and HRMS spectra of compound **14**.

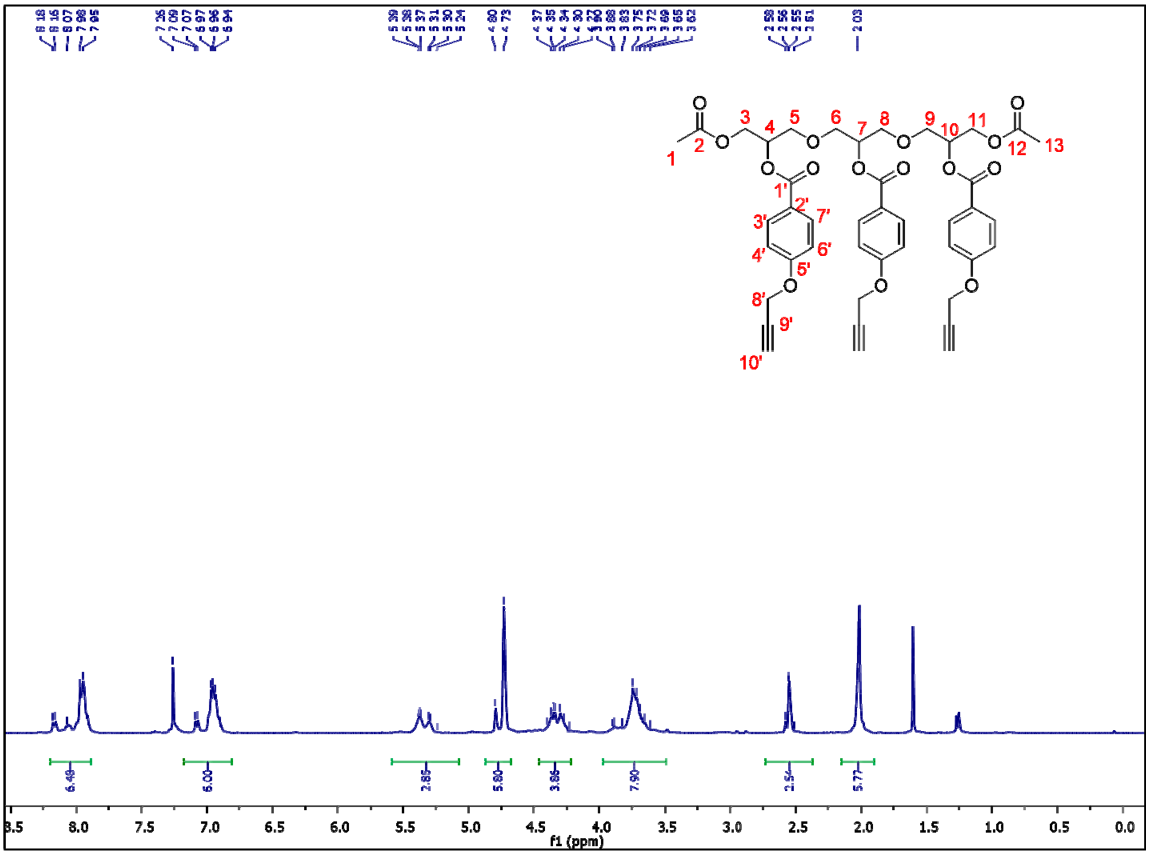
1
2
3
4
5
6
7
8
9
10
11
12
13
1'
2'
3'
4'
5'
6'
7'
8'
9'
10'
f1 (ppm)

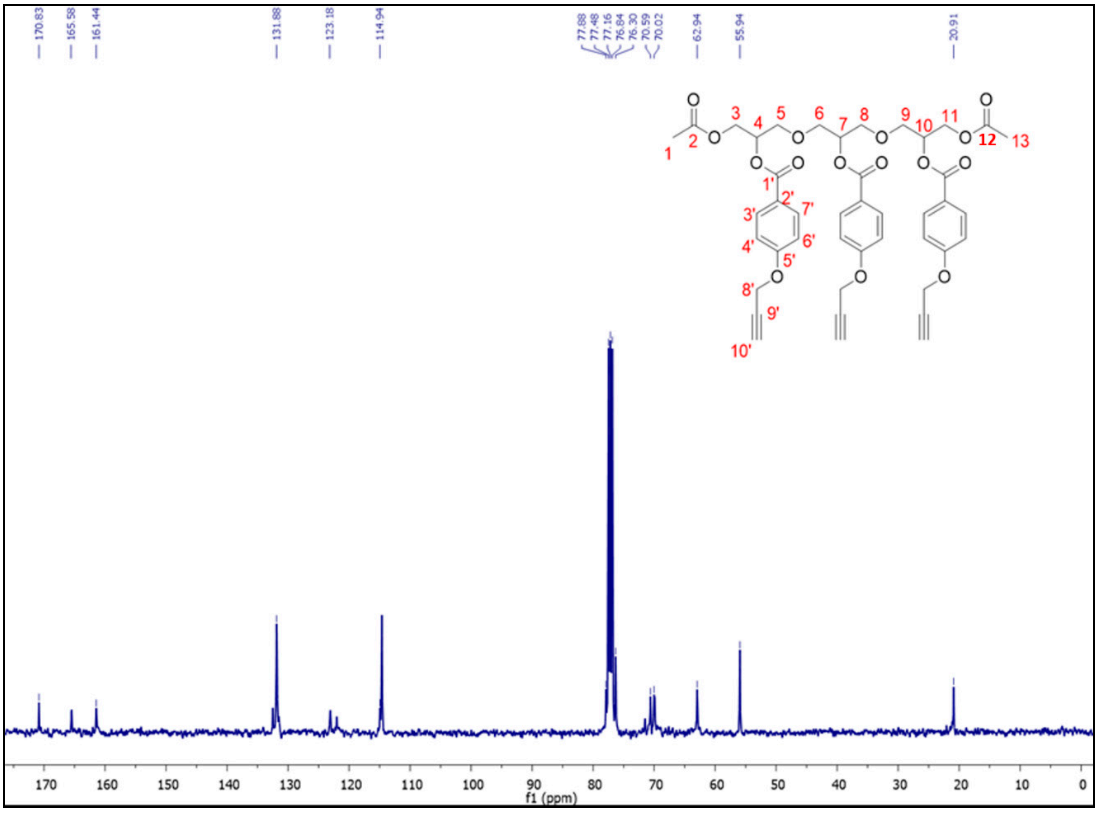
170.83
165.58
161.44
131.88
123.18
114.94
77.88
77.48
77.16
76.84
76.30
70.59
70.02
62.94
55.94
20.91
1
2
3
4
5
6
7
8
9
10
11
12
13
1'
2'
3'
4'
5'
6'
7'
8'
9'
10'
f1 (ppm)

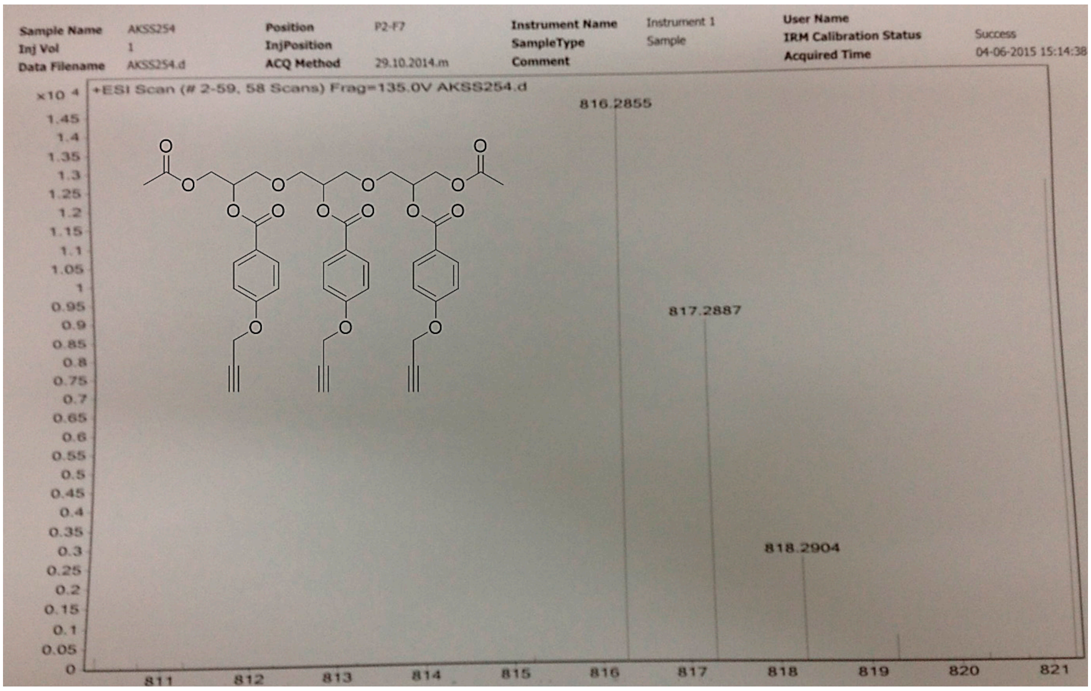

Figure S13. ^{1}H-, ^{13}C-NMR and HRMS spectra of compound **15**.

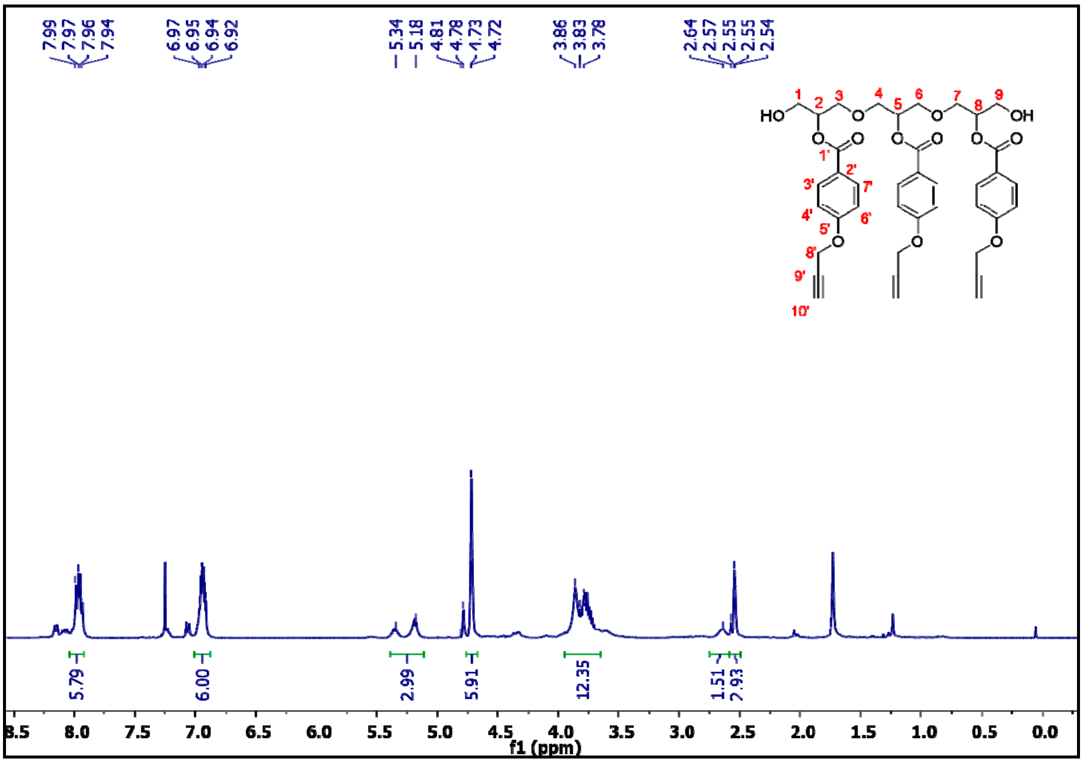

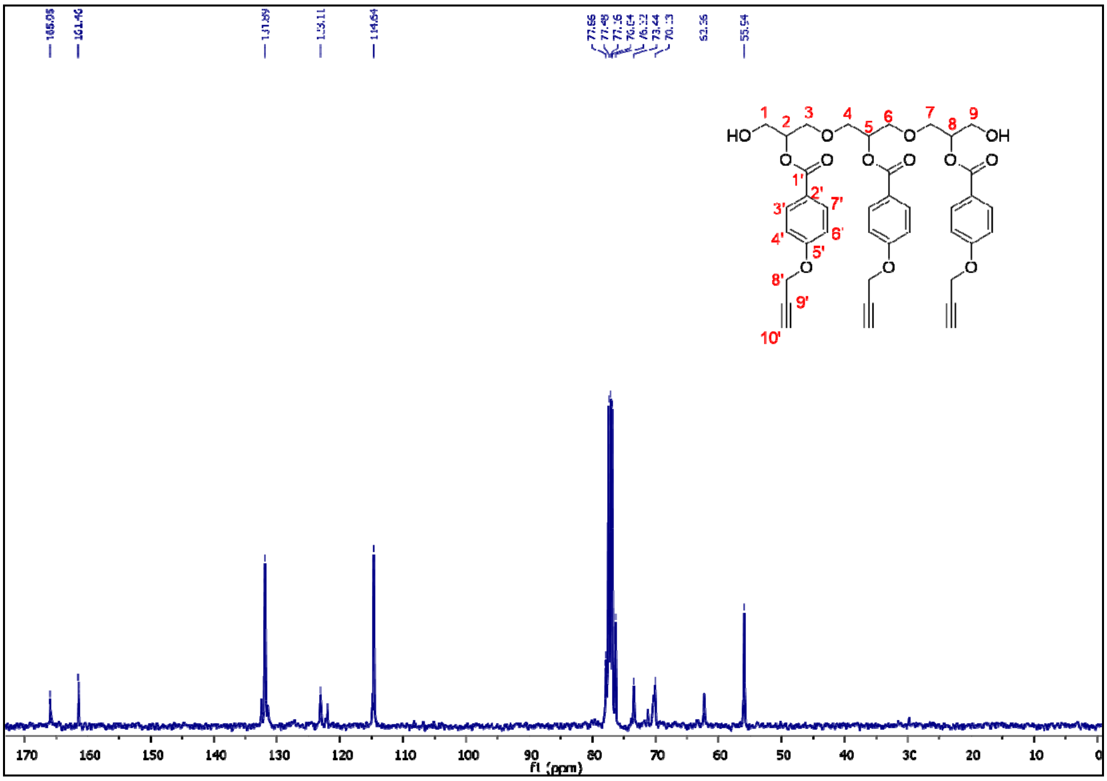

Compound Table

Compound Label	RT	Mass	Formula	MFG Formula	MFG Diff (ppm)	DB Formula
Cpd 2: C39 H38 O13	0.194	714.2303	C39 H38 O13	C39 H38 O13	1.26	C39 H38 O13

Compound Label	*m/z*	RT	Algorithm	Mass
Cpd 2: C39 H38 O13	737.2198	0.194	Find by Molecular Feature	714.2303

MFE MS Spectrum

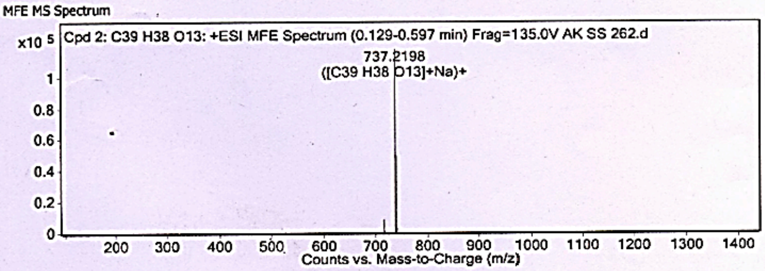

MFE MS Zoomed Spectrum

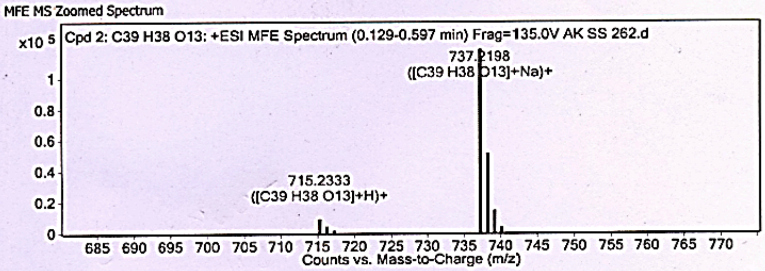

MS Spectrum Peak List

m/z	z	Abund	Formula	Ion
715.2333	1	8541.63	C39 H38 O13	(M+H)+
716.2388	1	3806.73	C39 H38 O13	(M+H)+
717.2389	1	1207.38	C39 H38 O13	(M+H)+
718.2367	1	225.08	C39 H38 O13	(M+H)+
737.2198	1	118139.77	C39 H38 O13	(M+Na)+
738.2228	1	49981.88	C39 H38 O13	(M+Na)+
739.2247	1	14280.19	C39 H38 O13	(M+Na)+
740.2267	1	3334.05	C39 H38 O13	(M+Na)+

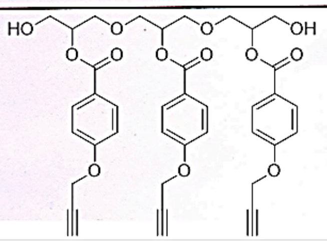

Figure S14. ^{1}H-, ^{13}C-NMR and HRMS spectra of compound **16**.
